# Folic acid for the primary prevention of stroke: a systematic review and meta-analysis

**DOI:** 10.3389/fnut.2024.1288417

**Published:** 2024-08-02

**Authors:** Jianjian Yang, Jia Wang, Bo Li, Yaxi Zhang

**Affiliations:** ^1^Department of Cardiology, Wenzhou Central Hospital, Wenzhou, Zhejiang, China; ^2^Department of Public Health, Weihai Maternal and Child Health Hospital, The Affiliated Weihai Second Municipal Hospital of Qingdao University, Weihai, Shandong, China; ^3^Zhangcun Town Health Center in Huancui District, Weihai, Shandong, China; ^4^Department of Neurology, Wenzhou Central Hospital, Wenzhou, Zhejiang, China

**Keywords:** folic acid, folate, supplementation, stroke, primary prevention, meta-analysis

## Abstract

**Objectives:**

Results from studies were inconsistent with regard to the effect of folic acid on the primary prevention of stroke. The aim of this study was to analyze the association between folic acid and the primary prevention of stroke using the data from observational studies and randomized controlled trials (RCTs).

**Methods:**

Eligible publications published until June 2024 were searched in the database of PubMed, Web of Science and Embase. This study included all observational studies and RCTs of folic acid with first stroke as the reporting endpoints. Relative risks (RRs) and 95% confidence intervals (CIs) were pooled in the random-effects model to assess the effect of folic acid on the primary prevention of stroke.

**Results:**

Results from 12 observational publications with 16 research, including 312,320 participants, were combined to explore the association between dietary folic acid intake and the primary prevention of stroke. The results showed that high dietary folic acid intake was associated with a 17% reduction in stroke incidence (RR:0.83; 95% CI: 0.73–0.94), and the effect of dietary folic acid was greater in areas without grain fortification (RR:0.80; 95% CI: 0.67–0.95). The pooled results from 12 RCTs, totaling 75,042 participants, indicated that folic acid supplementation was not associated with the stroke primary prevention (RR:0.92; 95% CI: 0.80–1.05), but folic acid supplementation was effective in areas without grain fortification (RR:0.78; 95% CI: 0.68–0.89).

**Conclusion:**

Our meta-analysis demonstrated that dietary folic acid is effective in stroke primary prevention, and folic acid supplementation is effective in stroke primary prevention only in areas without grain fortification.

**Systematic review registration:**

https://www.crd.york.ac.uk/PROSPERO/#myprospero, identifier CRD42024516991.

## 1 Introduction

Stroke is the leading cause of death and disability globally (1). There has been a twofold increase in the number of new strokes over the last 30 years, with around 795,000 strokes reported per year. On average, one person dies from a stroke every 3 min and 30 s (2, 3). Even those who survive a stroke have high rates of disability, requiring long-term rehabilitation and chronic care (4, 5). Therefore, the primary prevention of stroke is very important.

Among the range of preventive tactics, homocysteine (Hcy)-lowering medications have garnered significant interest, since research has indicated that Hcy may have an impact on stroke (6–8). Folic acid is crucial regulator in the metabolism of Hcy, and a shortage in folate can cause an accumulation of Hcy (9, 10). The efficacy of folic acid for stroke prevention has been debated for almost 30 years (11). Resent studies were designed to explored the relationship of dietary folic acid or folic acid supplements with stroke risk, and they found that both dietary folic acid and folic acid supplementation reduced the risk of stroke, but the combined results included primary and secondary prevention of stroke (11, 12). There is growing acknowledgment that there are major differences in the efficacy of folic acid for primary vs secondary prevention of stroke (13). The efficacy of folic acid for stroke primary prevention remains uncertain.

Several observational studies (9, 14–24) and RCTs (25–36) have investigated the link between folic acid and stroke primary prevention, however the findings were inconsistent. Of these, seven research (9, 14, 15, 17, 20, 21, 23) discovered an inverse relationship between dietary folic acid and the incidence of stroke, and one RCT demonstrated the protective effects of folic acid supplementation for stroke primary prevention. Another nine studies (14, 16–20, 22) and eleven RCTs (25–35) showed that folic acid was not associated with stroke incidence. Thus, we conducted a systematic review and meta-analysis to combine and synthesize the findings from the existing research.

## 2 Materials and methods

All analyses in this study were based on the guidelines of Preferred Reporting Items for Systematic reviews and Meta-Analyses (PRISMA) (37).

### 2.1 The literature search strategy

**FIGURE 1:**
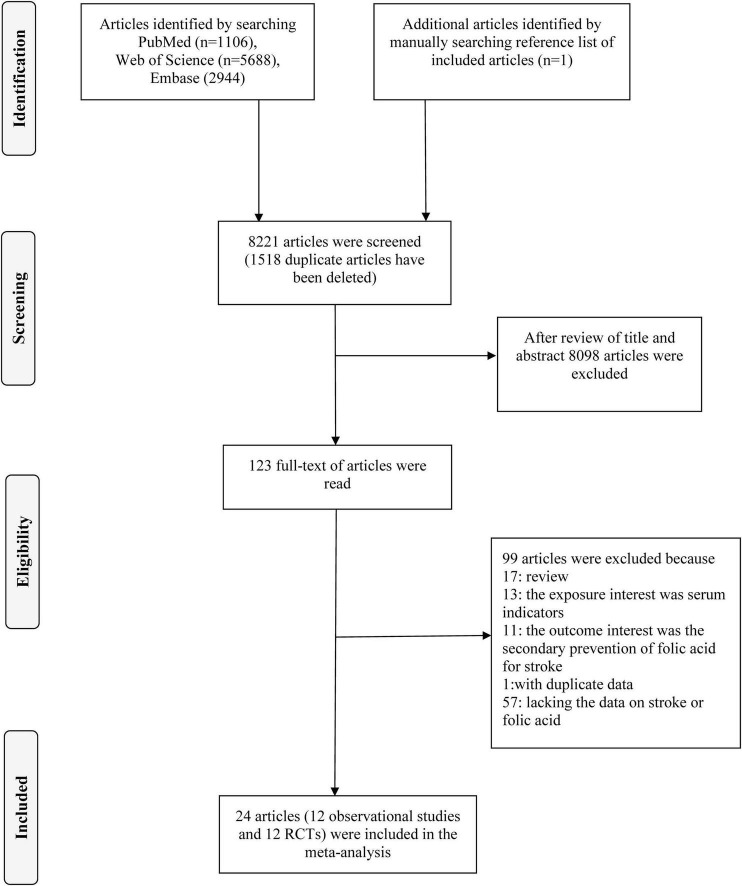
Flow chart of the selection of studies included in the meta-analysis.

We conducted a literature search in PubMed, web of science and Embase for relevant work published before June 2024. The search phrases used were “folic acid” (or “folate” or “folvite” or “vitamin B9” or “vitamin M”) and “Stroke” (or “cerebral infarction” or “intracranial hemorrhage” or “ischemic stroke” or “subarachnoid hemorrhage” or “cerebrovascular accident” or “brain ischemia” or “cerebral hemorrhage”). In addition, the references of the retrieved papers were manually examined for any relevant articles that may have been missed. The search approach was detailed in Figure 1 and Supplementary Tables 1, 2.

### 2.2 Inclusion criteria

The criteria for inclusion were as follows: (a) A published observational research or RCT in English. (b) The exposure interest in observational studies was the intake of dietary folic acid and the study intervention in RCTs included folic acid supplementation (with or without B vitamins and multivitamin supplementation). (c)The outcome interest was first stroke incidence. (d) Relative risks (RRs) and 95% confidence intervals (CIs) were shown. (e) The duration of the study intervention in the RCTs was at least 6 months. (f) For articles from the same population, the most comprehensive one would be selected.

Two investigators (Jianjian Yang and Jia Wang) meticulously searched and reviewed all studies separately. If two investigators disagreed on the eligibility of an article, a third reviewer will assess it to reach an agreement (Yaxi Zhang).

### 2.3 Data extraction

Two authors (Jianjian Yang and Bo Li) extracted the following information from each valuable article: first author’s name, publication year, study period, country in which the study was carried out, age range or mean age at baseline, RRs (RR is used to consistently represent all results for simplicity) with 95% CIs, type of stroke, grain fortification status. We furtherly gathered study design, method of assessing folic acid consumption, number of cases and sample size, adjustment factors in observational studies, and number of cases and participants in treatment and control groups, intervention regimen, type of control, duration of intervention in RCTs.

### 2.4 Quality assessment

The Newcastle-Ottawa Scale was utilized to evaluate the quality of the cohort studies and case-control articles in this investigation (38). The scale consisted of three components (selection, comparability, and outcome), with a maximum rating of 9 stars. All RCTs were assessed for the quality of randomization, blinding, withdrawals, random numbers generation, and concealment of allocation, and studies that took all five aspects into account were given a score of 5 (39).

### 2.5 Grain fortification status

We reviewed published papers on folic acid fortification policies for staple foods to understand the specifics of implementing mandated or voluntary fortification policies by June 2024 (40, 41). Folic acid fortified staple foods mainly include maize flour, rice, and wheat flour.

### 2.6 Statistical analyses

Most observational studies presented RR of stroke incidence for the highest vs. the lowest level of folic acid intake, so for studies (15, 19) with RR reported in the form of the lowest vs. the highest, the method of Hamling et al. (42) was used to convert risk estimates. We adopted the *I*^2^ to assess heterogeneity (43). The DerSimonian and Laird random effect model was utilized to combine study-specific RRs (95% CIs) (44). Subgroup analysis was used to examine the potential interactions. Meta-regression was performed to explore the potential origin of heterogeneity (45). Leave-one-out sensitivity analysis was conducted to evaluate the pivotal studies that have substantial impacts on between-study heterogeneity (46). The influence analysis assessed whether a single study had an obvious effect on the results. The Egger’s test and funnel plot were adopted to evaluate the publication bias (47).

For dose-response analysis, a 2-stage random-effects dose-response meta-analysis (48) was performed. First, a restricted cubic spline model with 3 knots at the 25th, 50th, and 75th percentiles (49) of the levels of dietary folic acid was estimated using generalized least square regression, taking into account the correlation within each set of published RRs (50). Second, the study-specific estimates were combined using the restricted maximum likelihood method in a multivariate random-effects meta-analysis (51). A *p* value for nonlinearity was calculated by testing the null hypothesis that the coefficient of the second spline is equal to 0.

We adopted Stata 15.0 to perform all analysis, and the results were considered statistically significant, when probabilities (*P*-values) reported 2-sided ≤ 0.05.

## 3 Results

### 3.1 Literature search and study characteristics

Following an exhaustive search and evaluation, 24 articles remained (9, 14–36), including 12 observational studies (9, 14–24) and 12 RCTs (25–36). Figure 1 and Supplementary Tables 1, 2 displayed the workflow for filtering accessible articles. In the observational studies, 16 results were pooled, because four publication (16, 20, 21, 24) containing two results, one for ischemic stroke and one for hemorrhagic stroke. Among the 12 studies, four (19, 21, 23, 24) with six studies were undertaken in North America, three (9, 15, 17) in Asia, and five (14, 16, 18, 20, 22) with seven studies were conducted in Europe (Table 1). Of these 12 RCTs, four (25, 27, 34, 35) were conducted in Europe, six (26, 29–33) in North America, one (28) in Oceania, and one (36) in Asia (Table 2).

**TABLE 1:** Summary of design characteristics in observational studies included in this meta-analysis.

References	Country (Study period)	Age range Mean age (Case/ control)	Study design	Method of assessing folate intake (Unit)	The exposure of folate intake Mean level or range	Sample (cases)	RR(95%CI)	Adjustment for Covariates	Types of stroke	Grain fortification
Zhang et al. (14)	Britain (2010–2018)	40–70	C-S	24-h ditery recall questionnaire (ug/day)	Quartile 1: 167.45	115664(1402)	1 (ref)	Age, sex, ethnic, physical activity, smoking status, employment, coffee, HDL-C, LDL-C, TC, adiposity	Total stroke	No
					Quartile 2: 221.52		0.94 (0.80–1.11)			
					Quartile 3: 321.41		0.90 (0.76–1.06)			
					Quartile 4: 432.81		0.86 (0.75–0.99)			
Van Guelpen et al. (16)	Sweden (1986–2000)	25–74	C-C-S	Food frequency questionnaire (mg/1000kcal/day)	Quartile 1	563 (139)	1 (ref)	BMI, current smoking, cholesterol, diabetes, hypertension, and plasma homocysteine	IS	No
					Quartile 2		1.14 (0.64–2.03)			
					Quartile 3		0.94 (0.48–1.8)			
					Quartile 4		1.19 (0.64–2.20)			
Van Guelpen et al. (16)	Sweden (1986–2000)	25–74	C-C-S	Food frequency questionnaire (mg/1000kcal/day)	Quartile 1	102 (25)	1 (ref)	BMI, current smoking, cholesterol, diabetes, hypertension, and plasma homocysteine	HS	No
					Quartile 2		0.13 (0.01–1.26)			
					Quartile 3		0.16 (0.02–1.21)			
					Quartile 4		0.16 (0.02–1.23)			
He et al. (21)	America (1986–2000)	40–75	C-S	Food frequency questionnaire (ug/day)	Quintile 1: 262	43732 (455)	1 (ref)	BMI, physical activity, history of hypertension and hypercholesterolemia, smoking status, aspirin use, alcohol, total calorie, intake of fiber, potassium, and vitamin E	IS	Yes
					Quintile 2: 336		1.00 (0.74–1.36)			
					Quintile 3: 413		0.75 (0.53–1.06)			
					Quintile 4: 547		0.96 (0.68–1.35)			
					Quintile 5: 821		0.66 (0.45–0.98)			
He et al. (21)	America (1986–2000)	40–75	C-S	Food frequency questionnaire (ug/day)	Quintile 1: 262	43732 (125)	1 (ref)	BMI, physical activity, history of hypertension and hypercholesterolemia, smoking status, aspirin use, alcohol, total calorie, intake of fiber, potassium, and vitamin E	HS	Yes
					Quintile 2: 336		1.28 (0.71–2.32)			
					Quintile 3: 413		1.49 (0.79–2.83)			
					Quintile 4: 547		1.31 (0.67–2.55)			
					Quintile 5: 821		0.86 (0.40–1.88)			
Weng et al. (15)	China (1990–1992)	40+	C–S	Food frequency questionnaire (ug/day)	<297.33	1772 (132)	1 (ref)	Age, sex, age*sex, hypertension, use of antihypertensive drugs, diabetes mellitus, area, central obesity, alcohol consumption habits, smoking habit, sex-smoking habit interaction, BMI, self-report heart disease, hypercholesterolemia, hypertriglyceridemia, physical activity, fibrinogen, apolipoprotein B, and plasminogen	IS	No
					297.33–369.45		1.15 (0.63–2.11)			
					>369.48		0.63 (0.40–0.98)			
Luu et al. (19)	America (1989–2005)	45–64	C-S	Food frequency questionnaire (ug/day)	Quartile 1: <155	12926 (594)	1 (ref)	Age, sex, current smoking status, diabetes, caloric intake, and hypertension	IS	Yes
					Quartile 2: 156–211		0.98 (0.68–1.43)			
					Quartile 3: 212–218		0.77 (0.54–1.12)			
					Quartile 4: ≥ 279		0.84 (0.64–1.10)			
Larsson et al. (20)	Finland (1985–2004)	50–69	C-S	Food frequency questionnaire (ug/day)	Quintile 1: 262	27111 (2702)	1 (ref)	Age, supplementation group, total number of cigarettes smoked daily, BMI, systolic and diastolic blood pressure, serum TC, serum HDL-C, histories of diabetes and coronary heart disease, leisure-time physical activity, and intakes of alcohol and total energy	IS	No
					Quintile 2: 300		0.99 (0.88–1.11)			
					Quintile 3: 330		1.09 (0.97–1.23)			
					Quintile 4: 360		0.98 (0.87–1.10)			
					Quintile 5: 410		0.80 (0.70–0.91)			
Larsson et al. (20)	Finland (1985–2004)	50–69	C-S	Food frequency questionnaire (ug/day)	Quintile 1: 262	27111 (579)	1 (ref)	Age, supplementation group, total number of cigarettes smoked daily, BMI, systolic and diastolic blood pressure, serum total cholesterol, serum HDL-C, histories of diabetes and coronary heart disease, leisure-time physical activity, and intakes of alcohol and total energy	HS	No
					Quintile 2: 300		1.16 (0.84–1.59)			
					Quintile 3: 330		0.90 (0.69–1.18)			
					Quintile 4: 360		0.98 (0.58–1.67)			
					Quintile 5: 410		0.93 (0.69–1.26)			
Choe et al. (9)	Korea (2011–2012)	65+	C-C-S	Food frequency questionnaire (ug/day)	<400	120 (60)	1 (ref)	Age, sex, smoking, TC, HDL-C, fasting blood glucose, hypertension, and regular exercise	IS	No
					≥ 400		0.16 (0.03-0.94)			
Bazzano et al. (23)	America (1971–1992)	25-74	C-S	24-h ditery recall questionnaire (ug/day)	Quartile 1: <136.0	9764 (926)	1 (ref)	Age, race, sex, systolic blood pressure, serum cholesterol, BMI, history of diabetes, physical activity, level of education, regular alcohol consumption, current cigarette smoking, saturated fat intake, and total energy intake	Total stroke	No
					Quartile 2: 136.0-203.7		0.93 (0.74–1.16)			
					Quartile 3: 203.7–300.6		0.85 (0.71–1.02)			
					Quartile 4: <300.6		0.80 (0.64–0.99)			
Al-Delaimy et al. (24)	America (1980–1998)	34–59	C-S	Food frequency questionnaire (ug/day)	Quintile 1: 30–210	83896 (924)	1 (ref)	Age, time period, smoking history, BMI, hormone use and menopausal status, currently taking aspirin, vitamin E supplements, physical activity, alcohol use, history of high blood pressure, history of diabetes, history of hypercholesterolemia, parental history of myocardial infarction, at or before the age of 65 years, total caloric intake	IS	Yes
					Quintile 2: 211–271		1.09 (0.89–1.33)			
					Quintile 3: 272–354		1.17 (0.95–1.44)			
					Quintile 4: 355–526		1.03 (0.81–1.30)			
					Quintile 5: <526		1.03 (0.80–1.33)			
Al-Delaimy et al. (24)	America (1980–1998)	34–59	C-S	Food frequency questionnaire (ug/day)	Quintile 1: 30–210	83896 (390)	1 (ref)	Age, time period, smoking history, BMI, hormone use and menopausal status, currently taking aspirin, vitamin E supplements, physical activity, alcohol use, history of high blood pressure, history of diabetes, history of hypercholesterolemia, parental history of myocardial infarction, at or before the age of 65 years, total caloric intake	HS	Yes
					Quintile 2: 211–271		1.29 (0.89–1.88)			
					Quintile 3: 272–354		0.98 (0.64–1.49)			
					Quintile 4: 355–526		1.23 (0.79–1.88)			
					Quintile 5: <526		1.05 (0.65–1.70)			
Park (17)	Korea (2007–2009)	57.4/57.9	C-C-S	Food frequency questionnaire (ug/day)	Quartile 1: ≤ 286.38	138 (69)	1 (ref)	Age, sex, BMI, and family history of stroke	Total stroke	No
					Quartile 2: 286.38–412.38		0.36 (0.12–1.10)			
					Quartile 3: 412.38–520.28		0.09 (0.02–0.39)			
					Quartile 4: <520.28		0.04 (0.008–0.23)			
Dalmeijer et al. (22)	the Netherlands (1997–2004)	49–70	C-S	Food frequency questionnaire (ug/day)	Quartile 1: ≤ 169	16165 (224)	1 (ref)	Hypertension, cholesterolemia, mean systolic blood pressure, age, total physical activity, BMI, smoking, diabetes, intake of energy, proteins, saturated fats, monounsaturated fats, polyunsaturated fats, alcohol, vitamin B2, vitamin B6, vitamin B12, betaine and choline.	Total stroke	No
					Quartile 2: 169–191		0.99 (0.64–1.54)			
					Quartile 3: 191–215		1.12 (0.66–1.89)			
					Quartile 4: <215		1.38 (0.67–2.81)			
Marniemi et al. (18)	Finland (1987–1997)	65–99	C-S	dietary history interviews (ug/day)	Tertile 1	660 (70)	1 (ref)	Age, gender, smoking, functional capacity and weight adjusted energy intake	Total stroke	No
					Tertile 2		0.83 (0.46–1.48)			
					Tertile 3		0.75 (0.38–1.46)			

RR, relative risk; CI, confidence interval; C-C-S, case-control study; C-S, cohort study; BMI, body mass index; HDL-C, high density lipoprotein-cholesterol; LDL-C, low density lipoprotein-cholesterol; TC, total cholesterol; IS, Ischemic stroke; HS, Hemorrhagic stroke.

**TABLE 2:** Summary of design characteristics in RCTs included in this meta-analysis.

References	Country (Study period)	Mean age	Number (Treatment/ control)	Cases (Treatment/ control)	Intervention regimen			Control	RR(95%CI)	Duration (months)	Grain fortifica-tion	Data quality
					Folic acid	Vitamin 12	Vitamin B6					
Ebbing et al. (25)	Norway (1999–2006)	61.4	1540/779	28/19	0.8mg/d	0.4mg/d	40mg/d	placebo	0.75 (0.42–1.33)	38.4	No	5
Wrone et al. (26)	America (1998–1999)	61.2	166/168	3/1	15mg/d	0.006mg/d	12.5mg/d	Folic acid 1 mg/d, vitamin B12 0.006 mg/d and vitamin B6 12.5 mg/d	3.04 (0.32–28.93)	24	Yes	3
Heinz et al. (27)	Germany (2002–2008)	61.0	327/323	11/15	5 mg/time three times a week	0.05 mg/time three times a week	20 mg/time three times a week	Folic acid 0.2 mg/time, vitamin B12 0.004 mg/time and vitamin B6 1 mg/time; three times a week	0.72 (0.34–1.55)	25.2	No	4
Zoungas et al. (28)	Australia; New Zealand (1998–2000)	57.0	156/159	8/18	15 mg/d	no	no	placebo	0.45 (0.20–1.01)	43.2	Partial	3
Bostom et al. (29)	America, Canada, Brazil (2002–2007)	52.0	2056/2054	35/32	5 mg/d	1 mg/d	50 mg/d	vitamin B12 0.002 mg/d, Vitamin B6 1.4 mg/d, folic acid 0 mg/d	1.09 (0.68–1.76)	48	Yes	3
Jamison et al. (30)	America (2001–2002)	65.8	1032/1024	37/41	40 mg/d	2 mg/d	100 mg/d	placebo	0.90 (0.58–1.39)	38.4	Yes	5
Albert et al. (31)	America (1998–2006)	62.8	2721/2721	79/69	2.5 mg/d	1 mg/d	50 mg/d	placebo	1.14 (0.83–1.57)	87.6	Yes	3
Sesso et al. (32)	America (1997–2011)	64.3	7317/7324	332/311	The intervention contains folic acid but the dosage of folic acid is unknown	The intervention contains vitamin B12 but the dosage of vitamin B12 is unknown	The intervention contains vitamin B6 but the dosage of vitamin B6 is unknown	placebo	1.07 (0.92–1.24)	134.4	Yes	4
Sesso et al. (33)	America (2015–2020)	72.1	10720/10720	121/116	The intervention contains folic acid but the dosage of folic acid is unknown	The intervention contains vitamin B12 but the dosage of vitamin B12 is unknown	The intervention contains vitamin B6 but the dosage of vitamin B6 is unknown	placebo	1.04 (0.81–1.34)	43.2	Yes	4
Righetti et al. (34)	Italy (2001–2003)	64.5	63/51	7/10	5 mg/d or 5 mg every other day	500 mg every other day	500 mg every other day	placebo	0.57 (0.23–1.39)	29.03	No	2
van Dijk et al. (35)	Netherlands (2008–2011)	74.0	1461/1458	46/60	0.4 mg/d	0.5 mg/d	no	placebo	0.77 (0.52–1.12)	24	No	5
Huo et al. (36)	China (2008–2013)	60.0	10348/10354	282/355	0.8 mg/d	no	no	Usual care	0.79 (0.68–0.93)	54	No	5

RCT, randomized controlled trial; RR, relative risk; CI, confidence interval.

### 3.2 Quality assessment

The mean score for case-control studies was 8.67 (range: 8 to 9) and for cohort studies was 8.00 (range: 6 to 9) after utilizing the Newcastle-Ottawa Scale to evaluate the quality of the 12 publications in this investigation. The mean score for RCTs was 3.83 (range: 3 to 5). The findings of the quality evaluation of the included studies were displayed in Supplementary Tables 3, 4 and Table 2.

### 3.3 The association between folic acid and the primary prevention of stroke

#### 3.3.1 The association between dietary folic acid and the primary prevention of stroke

**FIGURE 2:**
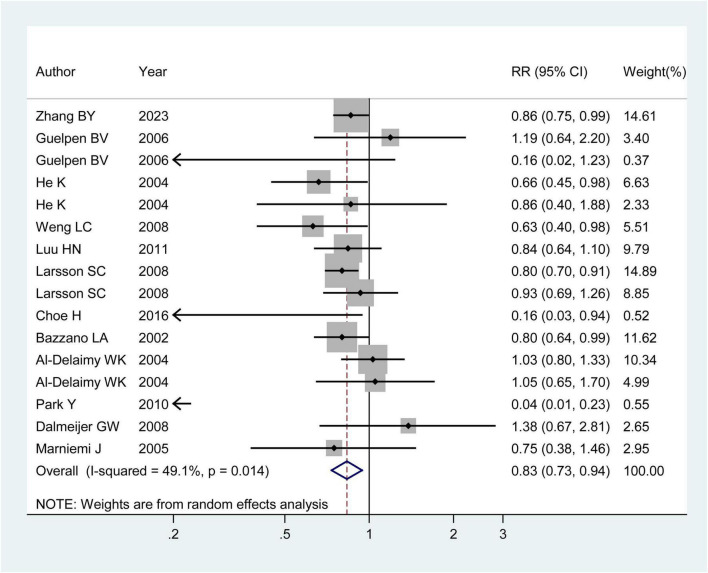
Forest plot of dietary folic acid and the primary prevention of stroke. The size of gray box is positively proportional to the weight assigned to each study, and horizontal lines represent the 95% confidence intervals. The RR (95% CI) in every article is the relative risk (95% confidence interval) of stroke incidence for the highest vs. the lowest stratification of dietary folic acid.

Twelve observational publications, which consisted of four case-control studies (9, 16, 17) and twelve cohort studies (14, 15, 18–24), were analyzed to explore the association between dietary folic acid and stroke primary prevention. These studies had 312,320 individuals and 8,816 stroke instances. Seven research (9, 14, 15, 17, 20, 21, 23) found a substantial link between dietary folic acid and stroke incidence, while nine studies (16, 18–22, 24) showed no connection. The combined RR (95%CI) of stroke incidence was 0.83 (95% CI: 0.73–0.94; *I^2^* = 49.10; *p_heterogeneity_* = 0.014; Figure 2), for the highest vs. lowest category of dietary folic acid intake. Seven research (9, 15, 16, 19–21, 24) examined the association between dietary folic acid and the occurrence of ischemic stroke, while four studies (16, 20, 21, 24) investigated the connection between dietary folic acid and hemorrhagic stroke. The pooled RRs (95%CIs) for the highest vs. lowest consumption categories of dietary folic acid were 0.82 (95% CI: 0.74–0.91; *I^2^* = 43.60%; *p_heterogeneity_* = 0.100) for ischemic stroke and 0.93 (95% CI: 0.73–1.18; *I^2^* = 2.90%; *p_heterogeneity_* = 0.378) for hemorrhagic stroke (Supplementary Figure 1).

The 12 observational studies were conducted in seven countries, and the background of grain folic acid fortification for each country is detailed in Supplementary Table 5. The integrated RRs (95%CIs) for the highest vs. the lowest category of dietary folic acid were 0.89 (95% CI: 0.76–1.05; *I^2^* = 4.10%; *p_heterogeneity_* = 0.383) for studies performed in areas with folic acid fortification and 0.80 (95% CI: 0.67–0.95; *I^2^* = 59.10%; *p_heterogeneity_* = 0.007) in areas without folic acid fortification (Supplementary Figure 2).

**FIGURE 3:**
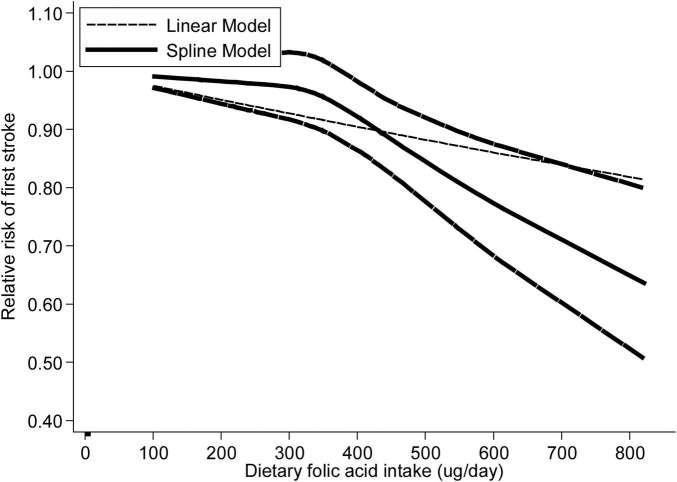
The dose-response analysis between dietary folic acid and the primary prevention of stroke with restricted cubic splines in a multivariate random-effects dose-response model. The solid line and the long dash line represent the estimated relative risks and its 95% CIs. Short dash line represents the linear relationship.

Analysis of nine articles (9, 14, 15, 17, 20–24) containing twelve research revealed a nonlinear relationship between folic acid consumption and stroke incidence (*p*_nonlinearity_ = 0.007) (Figure 3). The RRs with 95% CIs of stroke incidence were 0.99 (95% CI: 0.96–1.02), 0.97 (95% CI: 0.93–1.02), 0.92 (95% CI: 0.88–0.96), 0.82 (95% CI: 0.74–0.89) and 0.77 (95% CI: 0.68–0.87) for 241, 330, 410, 547 and 611 ug/day of dietary folic acid intake, respectively.

#### 3.3.2 The association between folic acid supplementation and the stroke primary prevention

**FIGURE 4:**
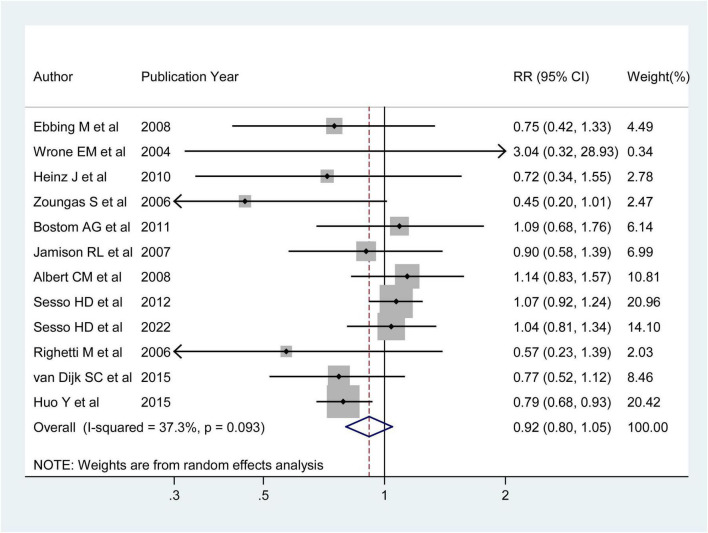
Forest plot of folic acid supplementation and the primary prevention of stroke. The size of gray box is positively proportional to the weight assigned to each study, and horizontal lines represent the 95% confidence intervals. The RR (95%CI) in every article is the relative risk (95% confidence interval) of stroke incidence for the highest vs. the lowest stratification of dietary folic acid.

Twelve RCTs were analyzed to explore the association between folic acid supplementation and the primary prevention of stroke. These studies included 75,042 individuals and 2,036 stroke cases. One study (36) reported an inverse association between folic acid supplementation and stroke incidence, while the other 11 studies (25–35) found no association between folic acid supplementation and stroke. The combined RR (95%CI) of stroke primary prevention was 0.92 (95% CI: 0.80–1.05; *I*^2^ = 37.30%; *p_heterogeneity_* = 0.093; Figure 4), for the highest vs. lowest category of folic acid supplementation. Of these 12 RCTs, four studies evaluated the association between folic acid supplementation and ischemic stroke incidence (26, 31, 32, 36), and three studies assessed the association between folic acid supplementation and hemorrhagic stroke incidence. The integrated RRs (95% CIs) for the highest vs. lowest categories of folic acid supplementation were 0.98 (95%CI: 0.74–1.29; *I*^2^ = 73.40%; *p_heterogeneity_* = 0.010) for ischemic stroke and 1.03 (95% CI: 0.80–1.34; *I^2^* = 0.00%; *p*_heterogeneity_ = 0.544) for hemorrhagic stroke (Supplementary Figure 3).

The 12 RCTs were conducted in nine countries. The pooled RRs (95%CIs) for the highest vs. the lowest category of supplementation were 1.06 (95% CI: 0.95–1.19; *I*^2^ = 0.00%; *p*_heterogeneity_ = 0.898) for studies performed in areas with folic acid fortification and 0.78 (95% CI: 0.68–0.89; *I^2^* = 0.00%; *p_heterogeneity_* = 0.968) in areas without folic acid fortification (Supplementary Figure 4).

### 3.4 Subgroup analysis

The subgroup results of dietary folic acid and primary prevention of stroke were shown in Table 3. In subgroup analysis stratified by geographic region, higher folic acid intake was significantly associated with decreased stroke incidence among studies conducted in Europe (OR: 0.85 95% CI: 0.77–0.95) and North America (OR: 0.86 95% CI: 0.76–0.98), but not in Asia (OR: 0.18 95% CI: 0.03–1.06). Increased folic acid intake was linked to a decreased risk of stroke in cohort studies (OR: 0.84 95% CI: 0.78–0.90), but not in case-control studies (OR: 0.21 95% CI: 0.04–1.23). With respect to major confounding factors, this inverse association remained after adjusting for BMI (OR: 0.84 95% CI: 0.70–0.99) and diabetes (OR: 0.86 95% CI: 0.77–0.97). Our analysis revealed that folic acid intake was inversely associated with stroke incidence in men (OR: 0.81 95% CI: 0.72–0.90), but not in women (OR: 1.06 95% CI: 0.86–1.32).

**TABLE 3:** Summary of RRs with 95% CIs for association between dietary folic acid and the primary prevention of stroke.

Stratification	Number of studies	OR (95% CI)	*I*^2^%	*P*_for heterogeneity_
All studies	16	0.83 (0.73–0.94)	49.10%	0.014
**Continent where the study was conducted**				
North America	6	0.86 (0.76–0.98)	0.00%	0.438
Asia	3	0.18 (0.03–1.06)	82.40%	0.003
Europe	7	0.85 (0.77–0.95)	10.80%	0.347
**Whether the results were adjusted for the history of hypertension or not**				
Yes	10	0.86 (0.70–1.05)	40.60%	0.087
No	6	0.81 (0.68–0.96)	63.70%	0.017
**Whether the results were adjusted for the history of diabetes or not**				
Yes	10	0.86 (0.77–0.97)	22.00%	0.240
No	6	0.60 (0.39–0.94)	71.60%	0.004
**Whether the results were adjusted for BMI or not**				
Yes	12	0.84 (0.70–0.99)	57.10%	0.007
No	4	0.83 (0.70–0.99)	20.40%	0.287
**The type of study design**				
Cohort studies	12	0.84 (0.78–0.90)	0.00%	0.568
Case-control studies	4	0.21 (0.04–1.23)	83.80%	<0.001
**Gender**				
Women	3	1.06 (0.86–1.32)	0.00%	0.752
Men	4	0.81 (0.72–0.90)	0.00%	0.589

RR, relative risk; CI, confidence interval.

The subgroup results of folic acid supplementation and primary prevention of stroke were shown in Table 4. We found that folic acid supplement intervention was significantly associated with decreased stroke incidence among studies conducted in Europe (OR: 0.85 95% CI: 0.77–0.95) and Asia (OR: 0.86 95% CI: 0.76–0.98), and it was not associated with stroke in any of the other subgroups.

**TABLE 4:** Summary of RRs with 95% CIs for association between folic acid supplementation and the primary prevention of stroke.

Stratification	Number of studies	RR (95% CI)	*I*^2^%	*P*_for heterogeneity_
All studies	12	0.92 (0.80–1.05)	37.30%	0.093
**Continent where the study was conducted**				
North America	6	1.06 (0.95–1.19)	0.00%	0.898
Europe	4	0.74 (0.56–0.97)	0.00%	0.946
Oceania	1	0.45 (0.20–1.01)	–	–
Asia	1	0.79 (0.68–0.92)	–	–
**Folic acid supplementation alone**				
Yes	2	0.69 (0.43–1.11)	44.10%	0.181
No	10	1.01 (0.91–1.12)	0.00%	0.562
**Folic acid dosage, mg/d**				
≤0.8	5	0.91 (0.77–1.08)	59.10%	0.044
<0.8	7	0.92 (0.71–1.18)	21.90%	0.262
**Duration of intervention, months**				
≤36	4	0.75 (0.55–1.03)	0.00%	0.600
<36	8	0.94 (0.81–1.10)	49.30%	0.055
**Data quality**				
<4	5	0.91 (0.62–1.34)	42.40%	0.139
≥4	5	0.91 (0.79–1.04)	41.20%	0.116

RR, relative risk; CI, confidence interval.

### 3.5 Meta-regression and sensitive analysis

A univariate meta-regression analysis was performed to investigate the origin of heterogeneity. The findings showed that whether adjusted for the history of hypertension (*p* = 0.730), the history of diabetes (*p* = 0.821), and BMI (*p* = 0.230), study design (*p* = 0.704), publication year (*p* = 0.896), and geographic region (*p* = 0.882) did not have a significant effect on the heterogeneity in the process of exploring the association between dietary folic acid and stroke primary prevention. In the process of exploring the association between folic acid supplementation and stroke primary prevention, geographic region (*p* = 0.928), data quality (*p* = 0.445), whether folic acid supplementation alone (*p* = 0.568), folic acid dosage (*p* = 0.615) and duration of intervention (*p* = 0.129) did not have a significant effect on the heterogeneity.

The leave-one-out sensitivity analysis revealed that the study conducted by Park (17) had a significant impact on the observed heterogeneity of the results pooled from observational studies. The between-study heterogeneity dropped to 16.96% after removal (*P _for heterogeneity_* = 0.264), and the OR (95%CI) was still significant with 0.84 (0.77–0.92). Considering the low heterogeneity of results synthesized from RCTs, we did not conduct leave-one-out sensitivity analysis.

### 3.6 Influence analysis and publication bias

**FIGURE 5:**
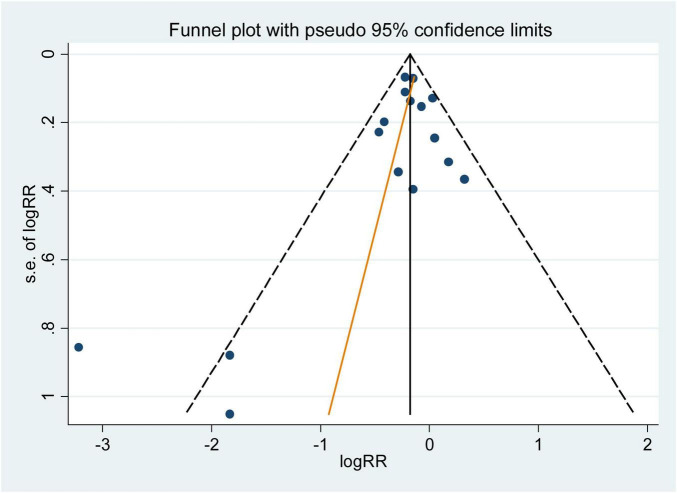
Funnel plot with pseudo 95% confidence limits for the analysis of dietary folic acid and he primary prevention of stroke. The RR (95%CI) in every article is the relative risk (95% confidence interval) of stroke incidence for the highest vs. the lowest stratification of dietary folic acid.

**FIGURE 6:**
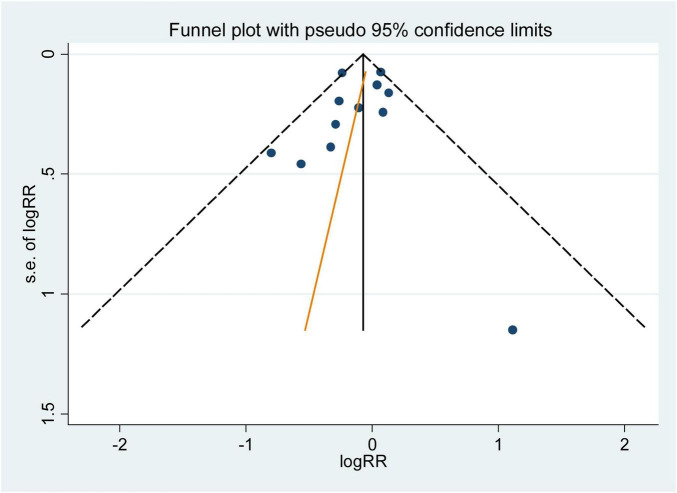
Funnel plot with pseudo 95% confidence limits for the analysis of folic acid supplementation and he primary prevention of stroke. The RR (95%CI) in every article is the relative risk (95% confidence interval) of stroke incidence for the highest vs. the lowest stratification of dietary folic acid.

The influence analysis showed that no observational study or RCT had a significant impact on the outcomes. No evidence of obvious small-study effect was found by the visual inspection of the funnel plot and Egger’s test in the included observational studies, with the p values of Egger’s test were 0.179 and 0.508 respectively. The results of funnel plot were displayed in Figures 5, 6.

## 4 Discussion

We included 24 studies in this meta-analysis, including 12 observational studies to assess the association between dietary folic acid intake and primary prevention of stroke, and 12 RCTs to assess the association between folic acid supplementation and primary prevention of stroke. The summaries from these studies disclosed that high dietary folic acid intake was associated with a 17% reduction in stroke incidence, and the effect of dietary folic acid was greater in areas without grain fortification. The negative link was significant for dietary folic acid and the occurrence of ischemic stroke, but not for hemorrhagic stroke. Moreover, folic acid supplementation was not associated with the stroke primary prevention, but folic acid supplementation was effective in areas without grain fortification.

The results of our meta-analysis aligned with those of other studies. An review of 22 randomized controlled trials revealed a 20% decrease in stroke risk and a 0.72umol/L decrease in Hcy levels in the group that took folic acid supplements compared to the control group (52). Two more studies (53, 54) also found a negative correlation between using folic acid supplements and the stroke events. The study showed that dietary folic acid and folic acid supplementation had a significant preventive effect on stroke in individuals without grain fortification, which aligned with other research indicating that folic acid supplement could decrease the risk of stroke, especially in regions without folic acid fortification (39, 55, 56). These results suggested that individuals with folic acid deficiency might gain more protection on stroke by increasing their dietary folic acid consumption or take folic acid supplements as appropriate. Encouraging the nutritional supplementation of folic acid through food should be advocated in countries where food is not fortified with folic acid due to its cost-effectiveness, safety, and wide applicability.

A negative association between folic acid and stroke incidence is biologically plausible. The mechanism by which folic acid reduces stroke incidence may be through its role as a substrate for the remethylation of Hcy to methionine, thereby reducing plasma Hcy concentration (57). Elevated amounts of Hcy can cause damage to blood vessels by accumulating endothelial cell toxicity and generating free radicals, which can contribute to the development of atherosclerosis (7, 58). Additionally, Hcy can enhance clotting function and disrupt the regulation of endothelium-dependent vasomotor (7). Folic acid not only regulates Hcy but also possesses antioxidant and vascular protecting properties, which are crucial in preventing stroke formation (59, 60).

Heterogeneity is prevalent in the meta-analysis, so it is principal to exploring the sources of heterogeneity. In this meta-analysis, *I^2^* = 49.10% from observational studies indicated moderate heterogeneity among the studies on the association between folic acid intake and stroke primary prevention. However, the P-values of all variables in the meta-regression model were greater than 0.05, and the source of heterogeneity could not be found through the meta-regression. By leaving one out, the result of sensitivity analysis indicated that one study (17) contributed a lot to heterogeneity. When this study was deleted, the heterogeneity of the results was reduced to 16.96%, and the results between dietary folic acid and stroke incidence were still significant, strengthening the stability and reliability of our results.

This meta-analysis had several strengths. First, we analyzed the association of dietary folic acid intake, folic acid supplementation with the primary prevention of stroke. Second, by enlarging the sample size and only including studies with first stroke as the reporting endpoints, we had a high statistical power to conclude the relationship between folic acid and the primary prevention of stroke. Third, we investigated the association between folic acid and the primary prevention of stroke in areas with and without acid fortification. Fourth, RRs (95% CIs) from observational studies were the results with adjusting for the most confounding factors, and we investigated the association of dietary folic acid with ischemic stroke and hemorrhagic stroke.

Several shortcomings need to be taken into account when interpreting the results. First, in the observational studies, results may have recall and selection biases, and the confounding factors adjusted in each article are different, so we could not completely eliminate the uncontrolled factors that may be inherent in these articles. Furthermore, the category of dietary folic acid is different, with some studies using four-point classifications and same using five-point classifications, which may underestimate the association between dietary folic acid and stroke. Second, in the RCTs, the dose of folic acid in the intervention group was slightly different, which may weaken the association between folic acid supplementation and stroke incidence. Third, populations with specific genetic backgrounds may respond differently to folic acid, but we don’t have enough data to analyze it. Finally, different methods of stroke diagnosis in the included studies may also have influenced the overall results.

## 5 Conclusion

The comprehensive review and meta-analysis indicated a substantial association between dietary folic acid intake and the primary prevention of stroke, and an association between folic acid supplementation and the primary prevention of stroke in areas without folic acid fortification. Our research indicated that increasing folic acid consumption in the diet or take folic acid supplements as appropriate can be an effective way in the primary prevention of stroke, particularly in countries that do not fortify foods with folic acid. This comprehensive analysis improved the current knowledge base and supplied pertinent data for clinical guidance and public health policies.

## Data availability statement

The original contributions presented in this study are included in this article/Supplementary material, further inquiries can be directed to the corresponding author.

## Author contributions

JY: Conceptualization, Data curation, Formal analysis, Writing–original draft. JW: Investigation, Project administration, Writing–review and editing. BL: Formal analysis, Software, Validation, Writing–review and editing. YZ: Methodology, Software, Validation, Writing–original draft.

## References

[1] GBD 2016 Causes of Death Collaborators. Global, regional, and national age-sex specific mortality for 264 causes of death, 1980-2016: A systematic analysis for the global burden of disease study 2016. *Lancet.* (2017) 390:1151–210. 10.1016/S0140-6736(17)32152-9 28919116 PMC5605883

[2] Tsao C Aday A Almarzooq Z Alonso A Beaton A Bittencourt M Heart disease and stroke statistics-2022 update: A report from the American heart association. *Circulation.* (2022) 145:e153–639. 35078371 10.1161/CIR.0000000000001052

[3] Wang Z Bu X Zhou B Li Y Nie Z. Dietary calcium intake and the risk of stroke: Meta-analysis of cohort studies. *Nutr Metab Cardiovasc Dis.* (2023) 33:934–46. 36958976 10.1016/j.numecd.2023.02.020

[4] Duncan P Bushnell C Sissine M Coleman S Lutz B Johnson A Comprehensive stroke care and outcomes: Time for a paradigm shift. *Stroke.* (2021) 52:385–93. 33349012 10.1161/STROKEAHA.120.029678

[5] Wang Z Chen B Zhou B Zhao D Wang L. Green tea consumption and the risk of stroke: A systematic review and meta-analysis of cohort studies. *Nutrition.* (2023) 107:111936. 10.1016/j.nut.2022.111936 36599267

[6] Mujumdar V Aru G Tyagi S. Induction of oxidative stress by homocyst(e)ine impairs endothelial function. *J Cell Biochem.* (2001) 82:491–500. 10.1002/jcb.1175 11500925

[7] Tsai J Perrella M Yoshizumi M Hsieh C Haber E Schlegel R Promotion of vascular smooth muscle cell growth by homocysteine: A link to atherosclerosis. *Proc Natl Acad Sci USA.* (1994) 91:6369–73. 10.1073/pnas.91.14.6369 8022789 PMC44203

[8] Hankey G Eikelboom J. Homocysteine and stroke. *Lancet.* (2005) 365:194–6. 15652586 10.1016/S0140-6736(05)17751-4

[9] Choe H Hwang J Yun J Kim J Song T Chang N Intake of antioxidants and B vitamins is inversely associated with ischemic stroke and cerebral atherosclerosis. *Nutr Res Pract.* (2016) 10:516–23. 10.4162/nrp.2016.10.5.516 27698959 PMC5037069

[10] Bazzano L Reynolds K Holder K He J. Effect of folic acid supplementation on risk of cardiovascular diseases: A meta-analysis of randomized controlled trials. *JAMA.* (2006) 296:2720–6. 17164458 10.1001/jama.296.22.2720

[11] Zhang N Zhou Z Chi X Fan F Li S Song Y Folic acid supplementation for stroke prevention: A systematic review and meta-analysis of 21 randomized clinical trials worldwide. *Clin Nutr.* (2024) 43:1706–16. 10.1016/j.clnu.2024.05.034 38824900

[12] Chen L Li Q Fang X Wang X Min J Wang F. Dietary intake of homocysteine metabolism-related b-vitamins and the risk of stroke: A dose-response meta-analysis of prospective studies. *Adv Nutr.* (2020) 11:1510–28. 10.1093/advances/nmaa061 32503038 PMC7666912

[13] Kleindorfer D Towfighi A Chaturvedi S Cockroft K Gutierrez J Lombardi-Hill D 2021 guideline for the prevention of stroke in patients with stroke and transient ischemic attack: A guideline from the American heart association/American stroke association. *Stroke.* (2021) 52:e364–467. 34024117 10.1161/STR.0000000000000375

[14] Zhang B Dong H Xu Y Xu D Sun H Han L. Associations of dietary folate, vitamin B6 and B12 intake with cardiovascular outcomes in 115664 participants: A large UK population-based cohort. *Eur J Clin Nutr.* (2023) 77:299–307. 10.1038/s41430-022-01206-2 36100703

[15] Weng L Yeh W Bai C Chen H Chuang S Chang H Is ischemic stroke risk related to folate status or other nutrients correlated with folate intake? *Stroke.* (2008) 39:3152–8. 18988909 10.1161/STROKEAHA.108.524934

[16] Van Guelpen B Hultdin J Johansson I Stegmayr B Hallmans G Nilsson T Folate, vitamin B12, and risk of ischemic and hemorrhagic stroke: A prospective, nested case-referent study of plasma concentrations and dietary intake. *Stroke.* (2005) 36:1426–31. 10.1161/01.STR.0000169934.96354.3a 15933256

[17] Park Y. Intakes of vegetables and related nutrients such as vitamin B complex, potassium, and calcium, are negatively correlated with risk of stroke in Korea. *Nutr Res Pract.* (2010) 4:303–10. 10.4162/nrp.2010.4.4.303 20827346 PMC2933448

[18] Marniemi J Alanen E Impivaara O Seppänen R Hakala P Rajala T Dietary and serum vitamins and minerals as predictors of myocardial infarction and stroke in elderly subjects. *Nutr Metab Cardiovasc Dis.* (2005) 15:188–97. 10.1016/j.numecd.2005.01.001 15955467

[19] Luu H Kingah P North K Boerwinkle E Volcik K. Interaction of folate intake and the paraoxonase Q192R polymorphism with risk of incident coronary heart disease and ischemic stroke: The atherosclerosis risk in communities study. *Ann Epidemiol.* (2011) 21:815–23. 10.1016/j.annepidem.2011.08.007 21982484 PMC3190162

[20] Larsson S Männistö S Virtanen M Kontto J Albanes D Virtamo J. Folate, vitamin B6, vitamin B12, and methionine intakes and risk of stroke subtypes in male smokers. *Am J Epidemiol.* (2008) 167:954–61. 10.1093/aje/kwm395 18270369

[21] He K Merchant A Rimm E Rosner B Stampfer M Willett W Folate, vitamin B6, and B12 intakes in relation to risk of stroke among men. *Stroke.* (2004) 35:169–74. 14671243 10.1161/01.STR.0000106762.55994.86

[22] Dalmeijer G Olthof M Verhoef P Bots M van der Schouw Y. Prospective study on dietary intakes of folate, betaine, and choline and cardiovascular disease risk in women. *Eur J Clin Nutr.* (2008) 62:386–94. 17375117 10.1038/sj.ejcn.1602725

[23] Bazzano L He J Ogden L Loria C Vupputuri S Myers L Dietary intake of folate and risk of stroke in US men and women: NHANES I epidemiologic follow-up study. National health and nutrition exaMINATION SURVey. *Stroke.* (2002) 33:1183–8. 10.1161/01.str.0000014607.90464.88 11988588

[24] Al-Delaimy W Rexrode K Hu F Albert C Stampfer M Willett W Folate intake and risk of stroke among women. *Stroke.* (2004) 35:1259–63. 15105514 10.1161/01.STR.0000127813.12854.9c

[25] Ebbing M Bleie Ø Ueland PM Nordrehaug JE Nilsen DW Vollset SE Mortality and cardiovascular events in patients treated with homocysteine-lowering B vitamins after coronary angiography: A randomized controlled trial. *JAMA.* (2008) 300:795–804. 10.1001/jama.300.7.795 18714059

[26] Wrone E Hornberger J Zehnder J McCann L Coplon N Fortmann S. Randomized trial of folic acid for prevention of cardiovascular events in end-stage renal disease. *J Am Soc Nephrol.* (2004) 15:420–6. 14747389 10.1097/01.asn.0000110181.64655.6c

[27] Heinz J Kropf S Domröse U Westphal S Borucki K Luley C B vitamins and the risk of total mortality and cardiovascular disease in end-stage renal disease: Results of a randomized controlled trial. *Circulation.* (2010) 121:1432–8. 20231532 10.1161/CIRCULATIONAHA.109.904672

[28] Zoungas S McGrath B Branley P Kerr P Muske C Wolfe R Cardiovascular morbidity and mortality in the atherosclerosis and folic acid supplementation trial (ASFAST) in chronic renal failure: A multicenter, randomized, controlled trial. *J Am Coll Cardiol.* (2006) 47:1108–16. 10.1016/j.jacc.2005.10.064 16545638

[29] Bostom A Carpenter M Kusek J Levey A Hunsicker L Pfeffer M Homocysteine-lowering and cardiovascular disease outcomes in kidney transplant recipients: Primary results from the folic acid for vascular outcome reduction in transplantation trial. *Circulation.* (2011) 123:1763–70. 10.1161/CIRCULATIONAHA.110.000588 21482964 PMC4887854

[30] Jamison R Hartigan P Kaufman J Goldfarb D Warren S Guarino P Effect of homocysteine lowering on mortality and vascular disease in advanced chronic kidney disease and end-stage renal disease: A randomized controlled trial. *JAMA.* (2007) 298:1163–70. 17848650 10.1001/jama.298.10.1163

[31] Albert C Cook N Gaziano J Zaharris E MacFadyen J Danielson E Effect of folic acid and B vitamins on risk of cardiovascular events and total mortality among women at high risk for cardiovascular disease: A randomized trial. *JAMA.* (2008) 299:2027–36. 10.1001/jama.299.17.2027 18460663 PMC2684623

[32] Sesso H Christen W Bubes V Smith J MacFadyen J Schvartz M Multivitamins in the prevention of cardiovascular disease in men: The physicians’ health study II randomized controlled trial. *JAMA.* (2012) 308:1751–60. 23117775 10.1001/jama.2012.14805 PMC3501249

[33] Sesso H Rist P Aragaki A Rautiainen S Johnson L Friedenberg G Multivitamins in the prevention of cancer and cardiovascular disease: The COcoa supplement and multivitamin outcomes study (COSMOS) randomized clinical trial. *Am J Clin Nutr.* (2022) 115:1501–10. 35294969 10.1093/ajcn/nqac056 PMC9170475

[34] Righetti M Serbelloni P Milani S Ferrario G. Homocysteine-lowering vitamin B treatment decreases cardiovascular events in hemodialysis patients. *Blood Purif.* (2006) 24:379–86. 16755160 10.1159/000093680

[35] van Dijk S Enneman A Swart K van Wijngaarden J Ham A Brouwer-Brolsma E Effects of 2-year vitamin B12 and folic acid supplementation in hyperhomocysteinemic elderly on arterial stiffness and cardiovascular outcomes within the B-PROOF trial. *J Hypertens.* (2015) 33:1897–906; discussion906. 10.1097/HJH.0000000000000647 26147383

[36] Huo Y Li J Qin X Huang Y Wang X Gottesman R Efficacy of folic acid therapy in primary prevention of stroke among adults with hypertension in China: The CSPPT randomized clinical trial. *JAMA.* (2015) 313:1325–35. 10.1001/jama.2015.2274 25771069

[37] Moher D Liberati A Tetzlaff J Altman D. Preferred reporting items for systematic reviews and meta-analyses: The PRISMA statement. *Int J Surg.* (2010) 8:336–41. 20171303 10.1016/j.ijsu.2010.02.007

[38] Ga Wells B O’Connell D Peterson J Welch V Losos M Tugwell P. The Newcastle–Ottawa scale (NOS) for assessing the quality if nonrandomized studies in meta-analyses. *ScienceOpen.* (2015).

[39] Zhao M Wu G Li Y Wang X Hou F Xu X Meta-analysis of folic acid efficacy trials in stroke prevention: Insight into effect modifiers. *Neurology.* (2017) 88:1830–8. 10.1212/WNL.0000000000003909 28404799

[40] Food Fortification Initiative. *Global progress of indus trially milled cereal grain fortification.* Haryana: Food Fortification Initiative (2024).

[41] Quinn M Halsey J Sherliker P Pan H Chen Z Bennett D Global heterogeneity in folic acid fortification policies and implications for prevention of neural tube defects and stroke: A systematic review. *EClinicalMedicine.* (2024) 67:102366. 10.1016/j.eclinm.2023.102366 38169713 PMC10758734

[42] Hamling J Lee P Weitkunat R Ambühl M. Facilitating meta-analyses by deriving relative effect and precision estimates for alternative comparisons from a set of estimates presented by exposure level or disease category. *Stat Med.* (2008) 27:954–70. 17676579 10.1002/sim.3013

[43] Higgins J Thompson S. Quantifying heterogeneity in a meta-analysis. *Stat Med.* (2002) 21:1539–58. 12111919 10.1002/sim.1186

[44] Higgins J Thompson S Deeks J Altman D. Measuring inconsistency in meta-analyses. *BMJ.* (2003) 327:557–60. 12958120 10.1136/bmj.327.7414.557 PMC192859

[45] Higgins J Thompson S. Controlling the risk of spurious findings from meta-regression. *Stat Med.* (2004) 23:1663–82. 15160401 10.1002/sim.1752

[46] Patsopoulos N Evangelou E Ioannidis J. Sensitivity of between-study heterogeneity in meta-analysis: Proposed metrics and empirical evaluation. *Int J Epidemiol.* (2008) 37:1148–57. 10.1093/ije/dyn065 18424475 PMC6281381

[47] Egger M Davey Smith G Schneider M Minder C. Bias in meta-analysis detected by a simple, graphical test. *BMJ.* (1997) 315:629–34. 9310563 10.1136/bmj.315.7109.629 PMC2127453

[48] Orsini N Li R Wolk A Khudyakov P Spiegelman D. Meta-analysis for linear and nonlinear dose-response relations: Examples, an evaluation of approximations, and software. *Am J Epidemiol.* (2012) 175:66–73. 10.1093/aje/kwr265 22135359 PMC3244608

[49] Harrell F Jr. Lee K Pollock B. Regression models in clinical studies: Determining relationships between predictors and response. *J Natl Cancer Inst.* (1988) 80:1198–202. 10.1093/jnci/80.15.1198 3047407

[50] Orsini N Bellocco R Greenland S. Generalized least squares for trend estimation of summarized dose-response data. *Stata J.* (2006) 6:40–57. 10.1177/1536867X060060010

[51] Jackson D White I Thompson S. Extending DerSimonian and Laird’s methodology to perform multivariate random effects meta-analyses. *Stat Med.* (2010) 29:1282–97. 10.1002/sim.3602 19408255

[52] Lan X Zhou Z Dang S Fan X Li D Su M Effect of supplementation with folic acid and B vitamins on cardiovascular outcomes: A meta-analysis of randomised controlled trials. *Lancet.* (2017) 390:83. 10.1371/journal.pone.0025142 21980387 PMC3182189

[53] Li Y Huang T Zheng Y Muka T Troup J Hu F. Folic acid supplementation and the risk of cardiovascular diseases: A meta-analysis of randomized controlled trials. *J Am Heart Assoc.* (2016) 5:e003768. 10.1161/JAHA.116.003768 27528407 PMC5015297

[54] Ye M Chen X Mao S Zhou J Liu M Wu Y. Effect of folic acid, vitamin B12, and B6 supplementation on the risk of cardiovascular and cerebrovascular diseases: An updated meta-analysis of randomized controlled trials. *Pteridines.* (2022) 33:39–48. 10.3760/cma.j.issn.0254-6450.2016.07.024 27453118

[55] Zeng R Xu C Xu Y Wang Y Wang M. The effect of folate fortification on folic acid-based homocysteine-lowering intervention and stroke risk: A meta-analysis. *Public Health Nutr.* (2015) 18:1514–21. 10.1017/S1368980014002134 25323814 PMC10271370

[56] Lee M Chiu S Saver J Hong K Wu Y Ovabiagele B. Folic acid therapy prevents stroke in countries without mandatory folic acid food fortification: A meta-analysis of randomized controlled trials. *Stroke.* (2016) 47:99–109. 10.5853/jos.2017.01522 29402063 PMC5836580

[57] McCully K. Vascular pathology of homocysteinemia: Implications for the pathogenesis of arteriosclerosis. *Am J Pathol.* (1969) 56:111–28. 10.1016/0002-8703(72)90051-8 5792556 PMC2013581

[58] Welch G Upchurch G Jr. Loscalzo J. Hyperhomocyst(e)inemia and atherothrombosis. *Ann N Y Acad Sci.* (1997) 811:48–58. 10.1111/j.1538-7836.2005.01364.x 9186584

[59] Wilmink H Stroes E Erkelens W Gerritsen W Wever R Banga J Influence of folic acid on postprandial endothelial dysfunction. *Arterioscler Thromb Vasc Biol.* (2000) 20:185–8. 10.3390/healthcare11182524 10634816

[60] Durand P Prost M Blache D. Pro-thrombotic effects of a folic acid deficient diet in rat platelets and macrophages related to elevated homocysteine and decreased n-3 polyunsaturated fatty acids. *Atherosclerosis.* (1996) 121:231–43. 10.1016/0021-9150(95)06724-8 9125297

