# Supplementary material for: Folic acid for the primary prevention of stroke: a systematic review and meta-analysis
Source: Front Nutr. 2024 Aug 2;11:1288417. doi: 10.3389/fnut.2024.1288417 (PMC11327562; doi:10.3389/fnut.2024.1288417)
Supplement: Supplementary file 1 [file Data_Sheet_1.docx]

Supporting Information

Table S1. The search strategy of folic acid and the primary prevention of stroke in PubMed, Web of Science and Embace

Table S2. Detailed process of literature exclusion

Table S3. Quality assessment of included cohort studies

Table S4. Quality assessment of included case-control studies

Table S5: Characteristics of folic acid fortification types by country and calendar year

Figure S1: Forest plot of dietary folic acid and the primary prevention of stroke grouped by stroke type

Figure S2: Forest plot of dietary folic acid and the primary prevention of stroke grouped by grain fortification

Figure S3: Forest plot of folic acid supplementation and the primary prevention of stroke grouped by stroke type

Figure S4: Forest plot of folic acid supplementation and the primary prevention of stroke grouped by grain fortification

**Table S1. The search strategy of folate intake and the primary prevention of stroke in PubMed, Web of Science and Embace**

| Step | Search term | NO.(articles) |
| --- | --- | --- |
| PubMed | | |
| #1 | Folate or folic acid or vitamin B9 or vitamin M or folvite | 71450 |
| #2 | Stroke or cerebral infarction or ischemic stroke or brain ischemia or cerebral hemorrhage or intracranial hemorrhage or subarachnoid hemorrhage or cerebrovascular accident | 610147 |
| #3=#1 and #2 | | 1106 |
| Web of Science | | |
| #4 | Folate or folic acid or vitamin B9 or vitamin M or folvite | 184189 |
| #5 | Stroke or cerebral infarction or ischemic stroke or brain ischemia or cerebral hemorrhage or intracranial hemorrhage or subarachnoid hemorrhage or cerebrovascular accident | 1138812 |
| #6=#4 and #5 | | 5688 |
| Embase | | |
| #7 | Folate or folic acid or vitamin B9 or vitamin M or folvite | 104877 |
| #8 | Stroke or cerebral infarction or ischemic stroke or brain ischemia or cerebral hemorrhage or intracranial hemorrhage or subarachnoid hemorrhage or cerebrovascular accident | 802809 |
| #9=#7 and #8 | | 2944 |
| PubMed＋Web of Science + Embase | | 9738 |

**Table S2 Detailed process of literature exclusion**

| Step | The number of articles before exclusion | The reasons of exclusion | NO.(articles) | The number of articles after exclusion | The number of references |
| --- | --- | --- | --- | --- | --- |
| 1 | 123 | Review | 17 | 106 | (1-17) |
| 2 | 106 | The exposure interest was serum indicators | 13 | 93 | (18-30) |
| 3 | 93 | The outcome interest was the secondary prevention of folic acid for stroke | 11 | 82 | (31-41) |
| 4 | 82 | With duplicate data | 1 | 81 | (42) |
| 5 | 81 | Lacking the data on stroke or folic acid | 57 | 24 | (43-99) |

**References**

1. Huang T, Chen Y, Yang B, Yang J, Wahlqvist ML, Li D. Meta-analysis of B vitamin supplementation on plasma homocysteine, cardiovascular and all-cause mortality. Clinical Nutrition. 2012;31(4):448-54.

2. Wang X, Qin X, Demirtas H, Li J, Mao G, Huo Y, et al. Efficacy of folic acid supplementation in stroke prevention: a meta-analysis. Lancet. 2007;369(9576):1876-82.

3. Bazzano LA, Reynolds K, Holder KN, He J. Effect of folic acid supplementation on risk of cardiovascular diseases: a meta-analysis of randomized controlled trials. Jama. 2006;296(22):2720-6.

4. Zhang C, Wang ZY, Qin YY, Yu FF, Zhou YH. Association between B vitamins supplementation and risk of cardiovascular outcomes: A cumulative meta-analysis of randomized controlled trials. PLoS ONE. 2014;9(9).

5. Zhou YH, Tang JY, Wu MJ, Lu J, Wei X, Qin YY, et al. Effect of folic acid supplementation on cardiovascular outcomes: a systematic review and meta-analysis. PLoS One. 2011;6(9):e25142.

6. Miller ER, 3rd, Juraschek S, Pastor-Barriuso R, Bazzano LA, Appel LJ, Guallar E. Meta-analysis of folic acid supplementation trials on risk of cardiovascular disease and risk interaction with baseline homocysteine levels. Am J Cardiol. 2010;106(4):517-27.

7. Jardine MJ, Kang A, Zoungas S, Navaneethan SD, Ninomiya T, Nigwekar SU, et al. The effect of folic acid based homocysteine lowering on cardiovascular events in people with kidney disease: systematic review and meta-analysis. Bmj. 2012;344:e3533.

8. Tian T, Yang KQ, Cui JG, Zhou LL, Zhou XL. Folic Acid Supplementation for Stroke Prevention in Patients With Cardiovascular Disease. American Journal of the Medical Sciences. 2017;354(4):379-87.

9. Lan X, Zhou Z, Dang S, Fan X, Li D, Su M, et al. Effect of supplementation with folic acid and B vitamins on cardiovascular outcomes: A meta-analysis of randomised controlled trials. The Lancet. 2017;390(SPEC.ISS 1):83.

10. Li Y, Huang T, Zheng Y, Muka T, Troup J, Hu FB. Folic Acid Supplementation and the Risk of Cardiovascular Diseases: A Meta-Analysis of Randomized Controlled Trials. Journal of the American Heart Association. 2016;5(8).

11. Zeng R, Xu CH, Xu YN, Wang YL, Wang M. The effect of folate fortification on folic acid-based homocysteine-lowering intervention and stroke risk: a meta-analysis. Public health nutrition. 2015;18(8):1514-21.

12. Lee M, Chiu SW, Saver JL, Hong KS, Wu YL, Ovabiagele B. Folic acid therapy prevents stroke in countries without mandatory folic acid food fortification: A meta-analysis of randomized controlled trials. Stroke. 2016;47.

13. Yang HT, Lee M, Hong KS, Ovbiagele B, Saver JL. Efficacy of folic acid supplementation in cardiovascular disease prevention: An updated meta-analysis of randomized controlled trials. European Journal of Internal Medicine. 2012;23(8):745-54.

14. Zhao M, Wu G, Li Y, Wang X, Hou FF, Xu X, et al. Meta-analysis of folic acid efficacy trials in stroke prevention: Insight into effect modifiers. Neurology. 2017;88(19):1830-8.

15. Ye M, Chen XH, Mao SF, Zhou J, Liu MF, Wu Y. Effect of folic acid, vitamin B12, and B6 supplementation on the risk of cardiovascular and cerebrovascular diseases: An updated meta-analysis of randomized controlled trials. Pteridines. 2022;33(1):39-48.

16. Spence JD, Yi Q, Hankey GJ. B vitamins in stroke prevention: time to reconsider. The Lancet Neurology. 2017;16(9):750-60.

17. Luo J, Duan W, Tan Y, Qu H, Shi D. Meta analysis of the effect of folic acid on primary prevention of h-type hypertensive stroke. Chinese Journal of Evidence-Based Medicine. 2020;20(10):1173-9.

18. Kelly PJ, Shih VE, Kistler JP, Barron M, Lee H, Mandell R, et al. Low vitamin B6 but not homocyst(e)ine is associated with increased risk of stroke and transient ischemic attack in the era of folic acid grain fortification. Stroke. 2003;34(6):e51-4.

19. Robinson K, Arheart K, Refsum H, Brattström L, Boers G, Ueland P, et al. Low circulating folate and vitamin B6 concentrations: risk factors for stroke, peripheral vascular disease, and coronary artery disease. European COMAC Group. Circulation. 1998;97(5):437-43.

20. Giles WH, Kittner SJ, Anda RF, Croft JB, Casper ML. Serum folate and risk for ischemic stroke. First National Health and Nutrition Examination Survey epidemiologic follow-up study. Cardiovascular therapeutics. 1995;26(7):1166-70.

21. Weikert C, Dierkes J, Hoffmann K, Berger K, Drogan D, Klipstein-Grobusch K, et al. B vitamin plasma levels and the risk of ischemic stroke and transient ischemic attack in a German cohort. Stroke. 2007;38(11):2912-8.

22. Kishida R, Yamagishi K. Serum folate and risk of disabling dementia: a community-based nested case-control study. 2023:1-7.

23. Zhang Z, Qin X, Spence JD, Li J, Zhang Y, Li Y, et al. Interaction of serum vitamin B(12) and folate with MTHFR genotypes on risk of ischemic stroke. BMC medicine. 2020;94(11):e1126-e36.

24. Carvalho JF, Shoenfeld Y. Low Folate Levels are Associated with Strokes and Less Venous Thromboses in Primary Antiphospholipid Syndrome. Mediterranean journal of rheumatology. 2022;33(4):478-9.

25. Xiong H, Li X, Cheng S, Chen P, Guo S, Huang X, et al. Folate Status and Mortality in US Adults With Diabetes: A Nationally Representative Cohort Study. Frontiers in cardiovascular medicine. 2022;9:802247.

26. Chen L, Wu C, Dong Z, Cao S, Ren N, Yan X. Methylenetetrahydrofolate reductase polymorphisms and elevated plasma homocysteine levels in small vessel disease. Brain and Behavior. 2023;13(5).

27. Medina MT, Sander L, Moncada S, Molina Y, Zablah I, Madrid M, et al. Hyperhomocystinemia, vitamin B12 and folate deficiencies and MTHFR mutations biomarkers in Hondurans stroke patients: A case-control study. Journal of the Neurological Sciences. 2023;455.

28. Preeti, Saini RP, Ambedkar SN, Saluja S. SerumVitamin B12 Levels in Patients of Ischaemic Stroke: A Cross-Sectional Study. Journal, Indian Academy of Clinical Medicine. 2023;24(3-4):188-92.

29. Xu J, Zhu X, Guan G, Zhang Y, Hui R, Xing Y, et al. Non-linear associations of serum and red blood cell folate with risk of cardiovascular and all-cause mortality in hypertensive adults. Hypertension Research. 2023;46(6):1504-15.

30. Zhang P, Xie X, Zhang Y. Associations between homocysteine and B vitamins and stroke: a cross-sectional study. Frontiers in Neurology. 2023;14.

31. Tang CY, Eshak ES, Shirai K, Tamakoshi A, Iso H. Associations of dietary intakes of vitamins B-1 and B-3 with risk of mortality from CVD among Japanese men and women: the Japan Collaborative Cohort study. British Journal of Nutrition. 2023;129(7):1213-20.

32. Cui R, Iso H, Date C, Kikuchi S, Tamakoshi A. Dietary folate and vitamin b6 and B12 intake in relation to mortality from cardiovascular diseases: Japan collaborative cohort study. Stroke. 2010;41(6):1285-9.

33. Lonn E, Yusuf S, Arnold MJ, Sheridan P, Pogue J, Micks M, et al. Homocysteine lowering with folic acid and B vitamins in vascular disease. N Engl J Med. 2006;354(15):1567-77.

34. Liem A, Reynierse-Buitenwerf GH, Zwinderman AH, Jukema JW, van Veldhuisen DJ. Secondary prevention with folic acid: results of the Goes extension study. Heart. 2005;91(9):1213-4.

35. Mark SD, Wang W, Fraumeni JF, Jr., Li JY, Taylor PR, Wang GQ, et al. Lowered risks of hypertension and cerebrovascular disease after vitamin/mineral supplementation: the Linxian Nutrition Intervention Trial. Am J Epidemiol. 1996;143(7):658-64.

36. B vitamins in patients with recent transient ischaemic attack or stroke in the VITAmins TO Prevent Stroke (VITATOPS) trial: a randomised, double-blind, parallel, placebo-controlled trial. Lancet Neurol. 2010;9(9):855-65.

37. Bønaa KH, Njølstad I, Ueland PM, Schirmer H, Tverdal A, Steigen T, et al. Homocysteine lowering and cardiovascular events after acute myocardial infarction. N Engl J Med. 2006;354(15):1578-88.

38. Toole JF, Malinow MR, Chambless LE, Spence JD, Pettigrew LC, Howard VJ, et al. Lowering homocysteine in patients with ischemic stroke to prevent recurrent stroke, myocardial infarction, and death: the Vitamin Intervention for Stroke Prevention (VISP) randomized controlled trial. Jama. 2004;291(5):565-75.

39. Lamas GA, Boineau R, Goertz C, Mark DB, Rosenberg Y, Stylianou M, et al. Oral high-dose multivitamins and minerals after myocardial infarction: a randomized trial. Ann Intern Med. 2013;159(12):797-805.

40. Armitage JM, Bowman L, Clarke RJ, Wallendszus K, Bulbulia R, Rahimi K, et al. Effects of homocysteine-lowering with folic acid plus vitamin B12 vs placebo on mortality and major morbidity in myocardial infarction survivors: a randomized trial. Jama. 2010;303(24):2486-94.

41. Galan P, Kesse-Guyot E, Czernichow S, Briancon S, Blacher J, Hercberg S. Effects of B vitamins and omega 3 fatty acids on cardiovascular diseases: a randomised placebo controlled trial. Bmj. 2010;341:c6273.

42. Shi Y, Zhang Z, Wang B, Wang Y, Kong X, Sun Y, et al. Effect of plateletcrit and methylenetetrahydrofolate reductase (MTHFR) C677T genotypes on folic acid efficacy in stroke prevention. Signal Transduct Target Ther. 2024;9(1):110.

43. Tang C, Eshak ES. Associations of dietary intakes of vitamins B(1) and B(3) with risk of mortality from CVD among Japanese men and women: the Japan Collaborative Cohort study. 2022;129(7):1-8.

44. Hariri M, Maghsoudi Z, Darvishi L, Askari G, Hajishafiee M, Ghasemi S, et al. B Vitamins and Antioxidants Intake is Negatively Correlated with Risk of Stroke in Iran. Int J Prev Med. 2013;4(Suppl 2):S284-9.

45. Zhang C, Chi FL, Xie TH, Zhou YH. Effect of B-vitamin supplementation on stroke: a meta-analysis of randomized controlled trials. PloS one. 2013;8(11):e81577.

46. Yuan S, Mason AM, Carter P, Burgess S, Larsson SC. Homocysteine, B vitamins, and cardiovascular disease: a Mendelian randomization study. 2021;19(1):97.

47. Yahn G, Bottiglieri T, Wasek B, Jadavji N. Vitamin B12 Deficiency Impairs Balance and Coordination After Photothrombotic Damage to the Sensorimotor Cortex in Adult Male and Female Mice After Ischemic Stroke. Stroke. 2021;52.

48. Preeti, Saini RP, Saluja S, Kabi BC. A Clinical study to determine levels of vitamin B12,Folic acid and Homocysteine in patients of Ischemic stroke. The Journal of the Association of Physicians of India. 2020;68(1):66.

49. Mbarek L, Sakka S, Moalla KS, Bouattour N, Daoud S, Hdiji O, et al. Hyperhomocysteinemia folate and vitamin B12 in young ischemic stroke young hyperhomocysteinemia folate and vitamin B12 in young ischemic stroke. Journal of the Neurological Sciences. 2021;429.

50. An P, Wan S, Luo Y, Luo J, Zhang X, Zhou S, et al. Micronutrient Supplementation to Reduce Cardiovascular Risk. Journal of the American College of Cardiology. 2022;80(24):2269-85.

51. Hankey GJ. B vitamins for stroke prevention. Stroke and vascular neurology. 2018;3(2):51-8.

52. Liu W, Cao ST, Shi D, Ye Z, Yu LL, Liang RY, et al. Daily folate consumption is associated with reduced all-cause and cardiovascular disease mortality among US adults with diabetes, prediabetes, or insulin resistance. Nutrition Research. 2023;114:71-80.

53. Kaplan RC, Williams-Nguyen JS, Huang YH, Mossavar-Rahmani Y, Yu B, Boerwinkle E, et al. Identification of Dietary Supplements Associated with Blood Metabolites in the Hispanic Community Health Study/Study of Latinos Cohort Study. Journal of Nutrition. 2023;153(5):1483-92.

54. Tan HK, Narasimhalu K, Ting SKS, Hameed S, Chang HM, De Silva DA, et al. B-vitamin supplementation on mitigating post-stroke cognition and neuropsychiatric sequelae: A randomized controlled trial. International Journal of Stroke. 2023;18(2):163-72.

55. Liu W, Ma XL, Gu HQ, Li H, Li ZX, Wang YJ. Elevated levels of total homocysteine after ischemic stroke: a potential marker for in-hospital outcomes. Neurological research. 2023;45(6):497-504.

56. Mbs GBY, Wasek B, Bottiglieri T, Malysheva O, Caudill MA, Jadavji NM. Dietary vitamin B12 deficiency impairs motor function and changes neuronal survival and choline metabolism after ischemic stroke in middle-aged male and female mice. 2023:1-10.

57. Hu F, Yu SC, Li J, Zhou W, Wang T, Huang X, et al. Association Between Hyperhomocysteinemia Combined with Metabolic Syndrome and Higher Prevalence of Stroke in Chinese Adults Who Have Elevated Blood Pressure. Medical Science Monitor. 2022;28.

58. Wei J, Ji JS. Modification of vitamin B6 on the associations of blood lead levels and cardiovascular diseases in the US adults. 2020;3(2):180-7.

59. Wei YP, Wang Z, He QQ, Siddiqi SM, Zhou ZY, Liu LS, et al. Inverse Association between Plasma Phylloquinone and Risk of Ischemic Stroke in Chinese Adults with Hypertension and High BMI: A Nested Case-Control Study. Journal of Nutrition. 2022;152(8):1927-35.

60. Wang Z, Ma H, Song Y, Lin TF, Liu LS, Zhou ZY, et al. Plasma selenium and the risk of first stroke in adults with hypertension: a secondary analysis of the China Stroke Primary Prevention Trial. American Journal of Clinical Nutrition. 2022;115(1):222-31.

61. Song Y, Li JY, Liu LS, Xu RCR, Zhou ZY, Xu BAM, et al. Plasma Vitamin E and the Risk of First Stroke in Hypertensive Patients: A Nested Case-Control Study. Frontiers in Nutrition. 2021;8.

62. Liu D, Wang JY, Xiao LJ, Gu SY, Ma Z, Zhou ZY, et al. Associations of plasma carnitine, lysine, trimethyllysine and glycine with incident ischemic stroke: Findings from a nested case-control study. Clinical Nutrition. 2022;41(9):1889-95.

63. Wei YP, Ma H, Xu BJM, Wang Z, He QQ, Liu LS, et al. Joint Association of Low Vitamin K1 and D Status With First Stroke in General Hypertensive Adults: Results From the China Stroke Primary Prevention Trial (CSPPT). Frontiers in Neurology. 2022;13.

64. Zhou F, Liu CZ, Ye LJ, Wang YK, Shao Y, Zhang GH, et al. The Relative Contribution of Plasma Homocysteine Levels vs. Traditional Risk Factors to the First Stroke: A Nested Case-Control Study in Rural China. Frontiers in Medicine. 2022;8.

65. Zhang JY, Zeng C, Huang X, Liao Q, Chen HS, Liu F, et al. Association of homocysteine and polymorphism of methylenetetrahydrofolate reductase with early-onset post stroke depression. Frontiers in Nutrition. 2022;9.

66. Pana TA, Dehghani M, Baradaran HR, Neal SR, Wood AD, Kwok CS, et al. Calcium intake, calcium supplementation and cardiovascular disease and mortality in the British population: EPIC-norfolk prospective cohort study and meta-analysis. European Journal of Epidemiology. 2021;36(7):669-83.

67. Kimura H, Yamagishi K, Muraki I, Tamakoshi A, Iso H. Prospective cohort study on potato intake and mortality from cardiovascular diseases: the Japan Collaborative Cohort Study (JACC study). European Journal of Nutrition. 2023;62(4):1859-66.

68. Che BZ, Zhong CK, Zhang RJ, Wang M, Zhang YH, Han LY. Multivitamin/mineral supplementation and the risk of cardiovascular disease: a large prospective study using UK Biobank data. European Journal of Nutrition. 2022;61(6):2909-17.

69. Park EJ. Association between vitamin B12 status and heart rate variability in patients with ischemic stroke. Medicine. 2023;102(16):e33428.

70. Guo LJ, Huang Y, Wan R, Shen Y, Hong K. Increased Blood Retinol Levels Are Associated With a Reduced Risk of TIA or Stroke in an Adult Population: Lifestyle Factors- and CVDs-Stratified Analysis. Frontiers in Cardiovascular Medicine. 2021;8.

71. Wang CY, Chen ZW, Zhang T, Liu J, Chen SH, Liu SY, et al. Elevated plasma homocysteine level is associated with ischemic stroke in Chinese hypertensive patients. European Journal of Internal Medicine. 2014;25(6):538-44.

72. Judd SE, Morgan CJ, Panwar B, Howard VJ, Wadley VG, Jenny NS, et al. Vitamin D deficiency and incident stroke risk in community-living black and white adults. International Journal of Stroke. 2016;11(1):93-102.

73. Dibaba DT, Xun PC, Fly AD, Bidulescu A, Tsinovoi CL, Judd SE, et al. Calcium Intake and Serum Calcium Level in Relation to the Risk of Ischemic Stroke: Findings from the REGARDS Study. Journal of Stroke. 2019;21(3):312-+.

74. Berghout BP, Fani L, Heshmatollah A, Koudstaal PJ, Ikram MA, Zillikens MC, et al. Vitamin D Status and Risk of Stroke The Rotterdam Study. Stroke. 2019;50(9):2293-8.

75. Li NF, Cai XT, Zhu Q, Yao XG, Lin MY, Gan L, et al. Association between Plasma Homocysteine Concentrations and the First Ischemic Stroke in Hypertensive Patients with Obstructive Sleep Apnea: A 7-Year Retrospective Cohort Study from China. Disease Markers. 2021;2021.

76. Nie J, Xie LL, Zhao BX, Li YB, Qiu BB, Zhu FX, et al. Serum Trimethylamine N-Oxide Concentration Is Positively Associated With First Stroke in Hypertensive Patients. Stroke. 2018;49(9):2021-8.

77. Murai U, Yamagishi K, Sata M, Kokubo Y, Saito I, Yatsuya H, et al. Seaweed intake and risk of cardiovascular disease: the Japan Public Health Center-based Prospective (JPHC) Study. American Journal of Clinical Nutrition. 2019;110(6):1449-55.

78. Ahmed S, Bogiatzi C, Hackam DG, Rutledge AC, Sposato LA, Khaw A, et al. Vitamin B-12 deficiency and hyperhomocysteinaemia in outpatients with stroke or transient ischaemic attack: a cohort study at an academic medical centre. BMJ open. 2019;9(1).

79. Zhang J, Cao J, Zhang H, Jiang C, Lin T, Zhou Z, et al. Plasma copper and the risk of first stroke in hypertensive patients: a nested case-control study. The American journal of clinical nutrition. 2019;110(1):212-20.

80. Yu YR, Zhang H, Song Y, Lin TF, Zhou ZY, Guo HY, et al. Plasma retinol and the risk of first stroke in hypertensive adults: a nested case-control study. American Journal of Clinical Nutrition. 2019;109(2):449-56.

81. Wang JC, Zhang XL, Zhang ZX, Zhang YY, Zhang JP, Li H, et al. Baseline Serum Bilirubin and Risk of First Stroke in Hypertensive Patients. Journal of the American Heart Association. 2020;9(12).

82. Vissers LET, Dalmeijer GW, Boer JMA, Verschuren WMM, van der Schouw YT, Beulens JWJ. Intake of Dietary Phylloquinone and Menaquinones and Risk of Stroke. Journal of the American Heart Association. 2013;2(6).

83. Rist PM, Cook NR, Manson JE, Buring JE, Rexrode KM. Effect of Vitamin D and Marine Omega-3 Fatty Acid Supplements on Stroke Outcomes. Stroke. 2020;51.

84. Ji W, Zhou H, Wang S, Cheng L, Fang Y. LOW SERUM LEVELS OF 25-HYDROXYVITAMIN D ARE ASSOCIATED WITH STROKE RECURRENCE AND POOR FUNCTIONAL OUTCOMES IN PATIENTS WITH ISCHEMIC STROKE. Journal of Nutrition Health & Aging. 2017;21(8):892-6.

85. Busch M, Scheidt-Nave C, Thiem U, Burghaus I, Trampisch HJ, Meves S, et al. ASSOCIATION OF LOW VITAMIN D LEVELS WITH INCREASED RISK OF STROKE IN OLDER ADULTS. Journal of Epidemiology and Community Health. 2011;65:A34-A.

86. Zhang ZY, Gu X, Tang Z, Guan SC, Liu HJ, Wu XG, et al. Homocysteine, hypertension, and risks of cardiovascular events and all-cause death in the Chinese elderly population: a prospective study. Journal of Geriatric Cardiology. 2021;18(10):796-808.

87. Yoshizaki T, Ishihara J, Kotemori A, Yamamoto J, Kokubo Y, Saito I, et al. Association of Vegetable, Fruit, and Okinawan Vegetable Consumption With Incident Stroke and Coronary Heart Disease. Journal of Epidemiology. 2020;30(1):37-45.

88. Yakoob MY, Shi PL, Hu FB, Campos H, Rexrode KM, Orav EJ, et al. Circulating biomarkers of dairy fat and risk of incident stroke in US men and women in 2 large prospective cohorts. American Journal of Clinical Nutrition. 2014;100(6):1437-47.

89. Virtanen JK, Voutilainen S, Happonen P, Alfthan G, Kaikkonen J, Mursu J, et al. Serum homocysteine, folate and risk of stroke: Kuopio Ischaemic Heart Disease Risk Factor (KIHD) Study. European journal of cardiovascular prevention and rehabilitation : official journal of the European Society of Cardiology, Working Groups on Epidemiology & Prevention and Cardiac Rehabilitation and Exercise Physiology. 2005;12(4):369-75.

90. Zhang Y, Liu D, Ma Z, Wang CC, Gu SJ, Zhou ZY, et al. Plasma beta-Alanine is Positively Associated With Risk of Ischemic Stroke: a Nested Case-Control Study. Journal of Nutrition. 2023;153(4):1162-9.

91. Wu S, Feng P, Li W, Zhuo S, Lu W, Chen P, et al. Dietary Folate, Vitamin B6, and Vitamin B12 and Risk of Cardiovascular Diseases among Individuals with Type 2 Diabetes: A Case-Control Study. Ann Nutr Metab. 2023;79(1):5-15.

92. Peng X, Gao Q, Zhou J, Ma J, Zhao D, Hao L. Association between dietary antioxidant vitamins intake and homocysteine levels in middle-aged and older adults with hypertension: a cross-sectional study. BMJ Open. 2021;11(10):e045732.

93. Jeon J, Park K. Dietary Vitamin B(6) Intake Associated with a Decreased Risk of Cardiovascular Disease: A Prospective Cohort Study. Nutrients. 2019;11(7).

94. Dalmeijer GW, Olthof MR, Verhoef P, Bots ML, van der Schouw YT. Prospective study on dietary intakes of folate, betaine, and choline and cardiovascular disease risk in women. Eur J Clin Nutr. 2008;62(3):386-94.

95. Assies JM, Sältz MD, Peters F, Behrendt CA, Jagodzinski A, Petersen EL, et al. Cross-Sectional Association of Dietary Patterns and Supplement Intake with Presence and Gray-Scale Median of Carotid Plaques-A Comparison between Women and Men in the Population-Based Hamburg City Health Study. Nutrients. 2023;15(6).

96. Haas CB, Su YR, Petersen P, Wang X, Bien SA, Lin Y, et al. Interactions between folate intake and genetic predictors of gene expression levels associated with colorectal cancer risk. Sci Rep. 2022;12(1):18852.

97. Gopinath B, Flood VM, Rochtchina E, Thiagalingam A, Mitchell P. Serum homocysteine and folate but not vitamin B12 are predictors of CHD mortality in older adults. Eur J Prev Cardiol. 2012;19(6):1420-9.

98. Xu X, Wei W, Jiang W, Song Q, Chen Y, Li Y, et al. Association of folate intake with cardiovascular-disease mortality and all-cause mortality among people at high risk of cardiovascular-disease. Clin Nutr. 2022;41(1):246-54.

99. Kishida R, Yamagishi K, Ikeda A, Hayama-Terada M, Shimizu Y, Muraki I, et al. Serum folate and risk of disabling dementia: a community-based nested case-control study. Nutr Neurosci. 2024;27(5):470-6.

Table S3. Quality assessment of included cohort studies

|  | Author (year) | | | | | | | | |
| --- | --- | --- | --- | --- | --- | --- | --- | --- | --- |
|  | Al-Delaimy WK et al (2004) | [Bazzano](https://pubmed.ncbi.nlm.nih.gov/?sort=date&size=200&term=Bazzano+LA&cauthor_id=11988588) LA et al (2002) | He K et al (2004) | Zhang BY et al (2023) | Weng LC et al (2008) | Luu HN et al (2011) | Larsson SC et al (2008) | Dalmeijer GW et al (2008) | Marniemi J et al (2005) |
| Selection |  |  |  |  |  |  |  |  |  |
| 1. Representativeness of the exposed cohort | 1 | 1 | 1 | 1 | 1 | 1 | 1 | 1 | 1 |
| 1. Selection of the non exposed cohort | 1 | 1 | 1 | 1 | 1 | 1 | 1 | 1 | 1 |
| 1. Ascertainment of exposure | 0 | 0 | 1 | 1 | 1 | 0 | 0 | 0 | 0 |
| 1. Outcome of interest not present at start of study | 1 | 1 | 1 | 1 | 1 | 1 | 1 | 1 | 0 |
| Comparability |  |  |  |  |  |  |  |  |  |
| 1. Comparability of cohorts on the bias of the design or analysis^1^ | 2 | 2 | 2 | 2 | 2 | 1 | 2 | 2 | 1 |
| Outcome |  |  |  |  |  |  |  |  |  |
| 1. Assessment of outcome | 1 | 1 | 1 | 1 | 1 | 1 | 1 | 1 | 1 |
| 1. Follow-up long enough for outcomes to occur^2^ | 1 | 1 | 1 | 1 | 1 | 1 | 1 | 1 | 1 |
| 1. Adequacy of follow-up of cohorts^3^ | 1 | 1 | 1 | 1 | 1 | 1 | 1 | 1 | 1 |
| Overall quality score | 8 | 8 | 9 | 9 | 9 | 7 | 8 | 8 | 6 |

The quality of studies was assessed by the Newcastle-Ottawa quality assessment scale.

^1^A maximum of 2 scores could be awarded for this item. Studies that controlled for age or gender received 1 score, whereas studies that controlled for other important confounders received 1 additional score.

^2^A cohort study with a follow-up time ＞3 years was assigned 1 score.

^3^A cohort study with a follow-up rate ＞70% was assigned 1score.

A study can be awarded a maximum score of 9 in total.

Table S4. Quality assessment of included case-control studies

|  | Author (year) | | |
| --- | --- | --- | --- |
|  | Choe H et al (2016)^14^ | [Park](https://pubmed.ncbi.nlm.nih.gov/?sort=date&size=200&term=Park+Y&cauthor_id=20827346) Y  et al (2010)^19^ | Guelpen BV et al (2006) |
| Selection |  |  |  |
| 1. The case definition is adequate. | 1 | 1 | 1 |
| 1. Representativeness of the cases.案例的代表性。 | 1 | 1 | 1 |
| 1. Selection of controls. | 1 | 1 | 1 |
| 1. Definition of controls is adequate. | 1 | 1 | 1 |
| Comparability |  |  |  |
| 1. Comparability of cases and controls on the basis of the design or analysis基于设计或分析的案例和对照的可比性 | 2 | 2 | 2 |
| Outcome |  |  |  |
| 1. Ascertainment of Exposure. | 1 | 1 | 1 |
| 1. Same method of ascertainment for cases and controls | 1 | 1 | 1 |
| 1. Non-Response Rate | 1 | 1 | 0 |
| Overall quality score | 9 | 9 | 8 |

The quality of studies was assessed by the Newcastle-Ottawa quality assessment scale.

Table S5: Characteristics of folic acid fortification types by country and calendar year

| Country | Food Vehicle | Fortification status | Nutrient level in standard (mg/kg) | Year of Legislation |
| --- | --- | --- | --- | --- |
| America | Rice | Mandatory fortification | 1.87 | 1996 |
| America | Wheat Flour | Mandatory fortification | 1.54 | 1996 |
| Australia | Wheat Flour | Mandatory fortification | 2.5 | 2009 |
| Britain | - | Voluntary fortification | 0.3 | 2006 |
| Brazil | Wheat Flour | Mandatory fortification | 1.8 | 2002 |
| Canada | Wheat flour, Enriched Uncooked Pasta | Mandatory fortification | 1.5 | 1998 |
| China | Wheat flour, Rice | Voluntary fortification | 2.0 | 2012 |
| Finland | - | Voluntary Fortification | 0.3 | 2006 |
| Italy | - | Voluntary fortification | 0.3 | 2006 |
| Germany | - | Voluntary Fortification | 0.3 | 2006 |
| Korea | - | None | - | - |
| Norway | - | Voluntary Fortification | 0.3 | 2006 |
| Sweden | - | Voluntary fortification | 0.3 | 2006 |
| The Netherlands | - | Voluntary fortification | 0.3 | 2006 |





Figure S 1 Forest plot of dietary folic acid and the primary prevention of stroke grouped by stroke type





Figure S 2 Forest plot of dietary folic acid and the primary prevention of stroke grouped by grain fortification


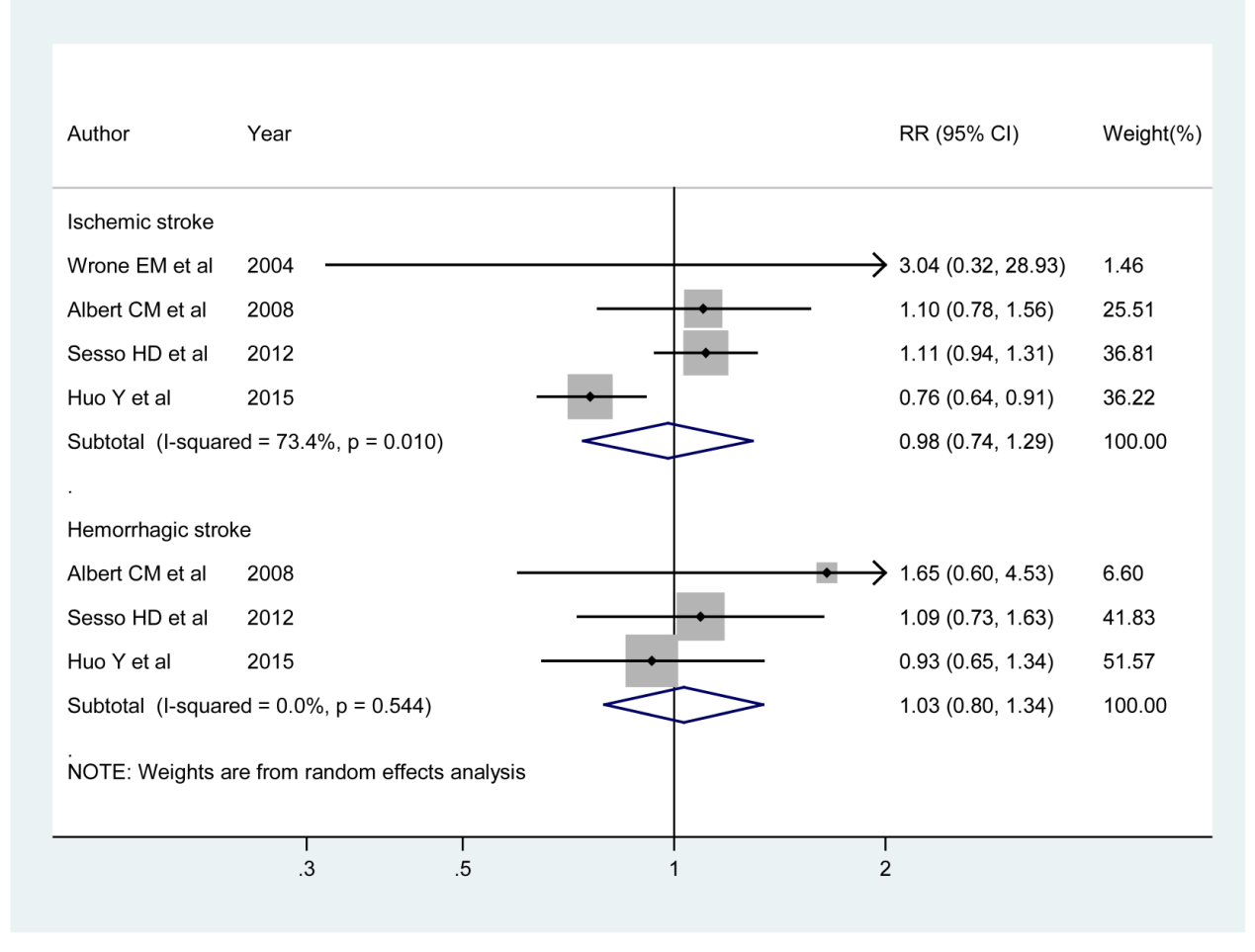


Figure S 3 Forest plot of folic acid supplementation and the primary prevention of stroke grouped by stroke type


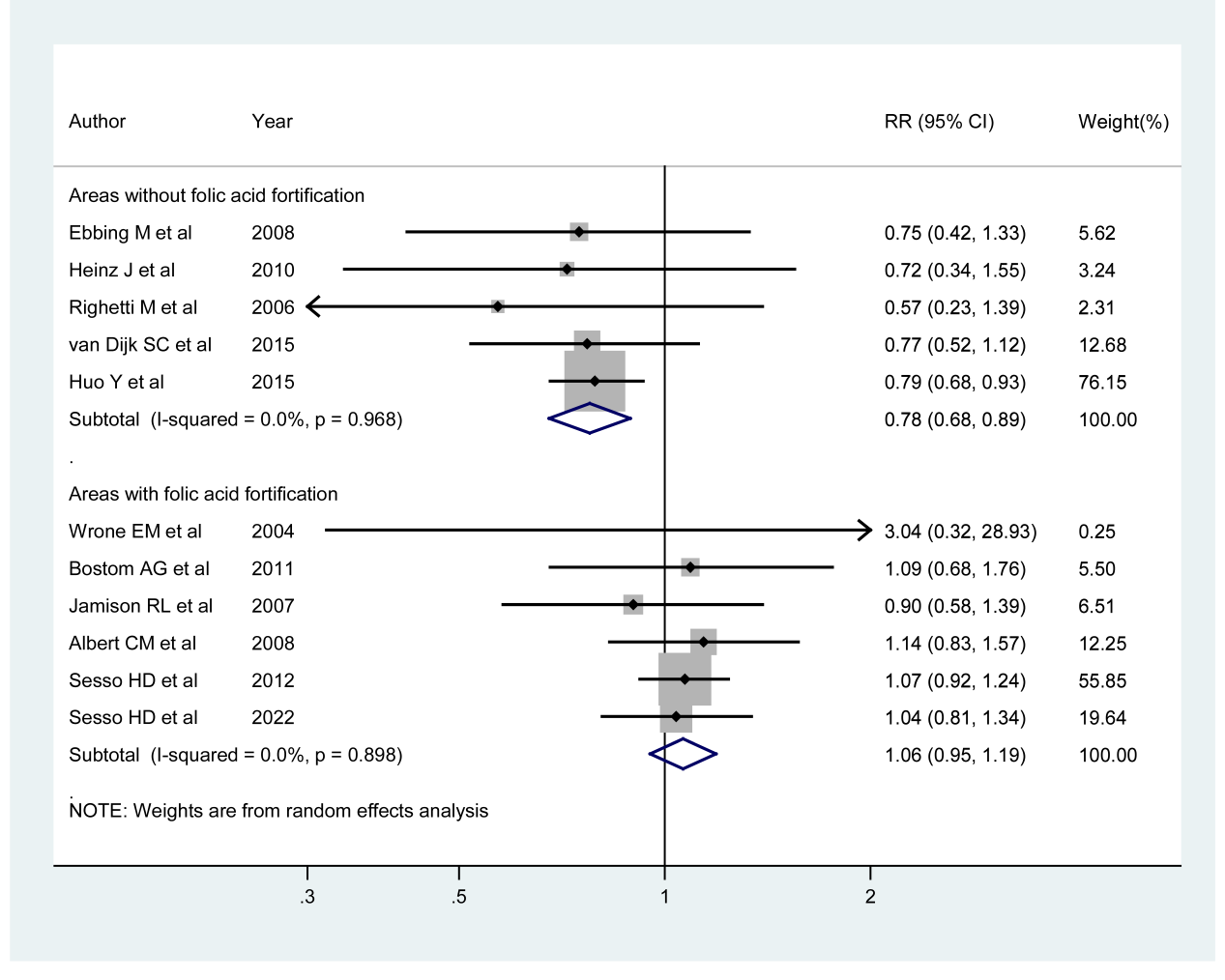


Figure S 4 Forest plot of folic acid supplementation and the primary prevention of stroke grouped by grain fortification
